# Beyond the visible: metal-ion-doped inorganic UV phosphors for advanced photonics

**DOI:** 10.1038/s41377-026-02276-8

**Published:** 2026-05-06

**Authors:** Yi Zhang, Yanjie Liang, Feng Liu, Xiao-Jun Wang

**Affiliations:** 1 School of Materials Science & Engineering, Shandong University, Jinan, China; 2 State Key Laboratory of Coatings for Advanced Equipment, Shandong University, Jinan, China; 3 Key Laboratory for UV-Emitting Materials and Technology of Ministry of Education, Northeast Normal University, Changchun, China; 4 Department of Physics, Georgia Southern University, Statesboro, GA USA

**Keywords:** Lasers, LEDs and light sources, Optical materials and structures, Electronics, photonics and device physics

## Abstract

Inorganic ultraviolet (UV) luminescent materials doped with metal ions (including rare-earth and heavy main-group metal ions) exhibit distinctive electronic transitions, excellent photostability, and tunable emission characteristics, making them highly promising for applications in optoelectronics, environmental remediation, and biomedicine. Recent progress in metal-ion-doped UV-emitting systems, such as persistent luminescence, upconversion luminescence, and mechanoluminescence, has significantly expanded the possibilities for UV light generation and utilization. This review provides a comprehensive and systematic overview of the luminescence mechanisms, recent advances, and emerging applications of these materials, structured according to dominant luminescence modes and supplemented by spectral classifications. First, UV persistent luminescence materials are critically examined with emphasis on optimization strategies and future research opportunities. Next, the UV upconversion luminescence systems are reviewed, highlighting mechanistic insights and breakthrough achievements. The discussion then turns to UV mechanoluminescent materials, focusing on their ability to emit light under mechanical stimulation and on recent progress in material design and device integration. Applications of these metal-ion-doped inorganic UV phosphors across different spectral regions are further analyzed under the guiding principle that “performance dictates application”. Finally, key challenges and future directions are outlined to provide a forward-looking perspective for advancing inorganic UV luminescent materials.

## Abstract (web summary)

This review provides a comprehensive and systematic overview of the luminescence mechanisms, recent advances, and emerging applications of metal-ion-doped inorganic UV luminescent materials.

## Graphical abstract

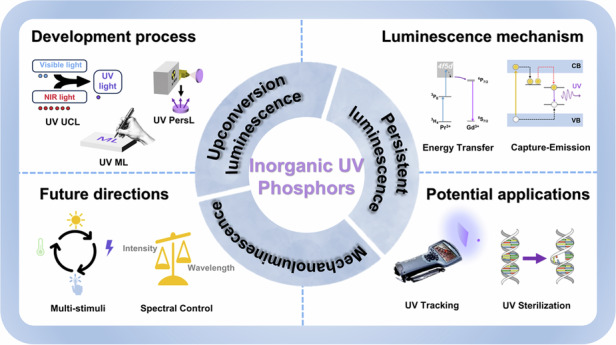

## Introduction

**Fig. 1:**
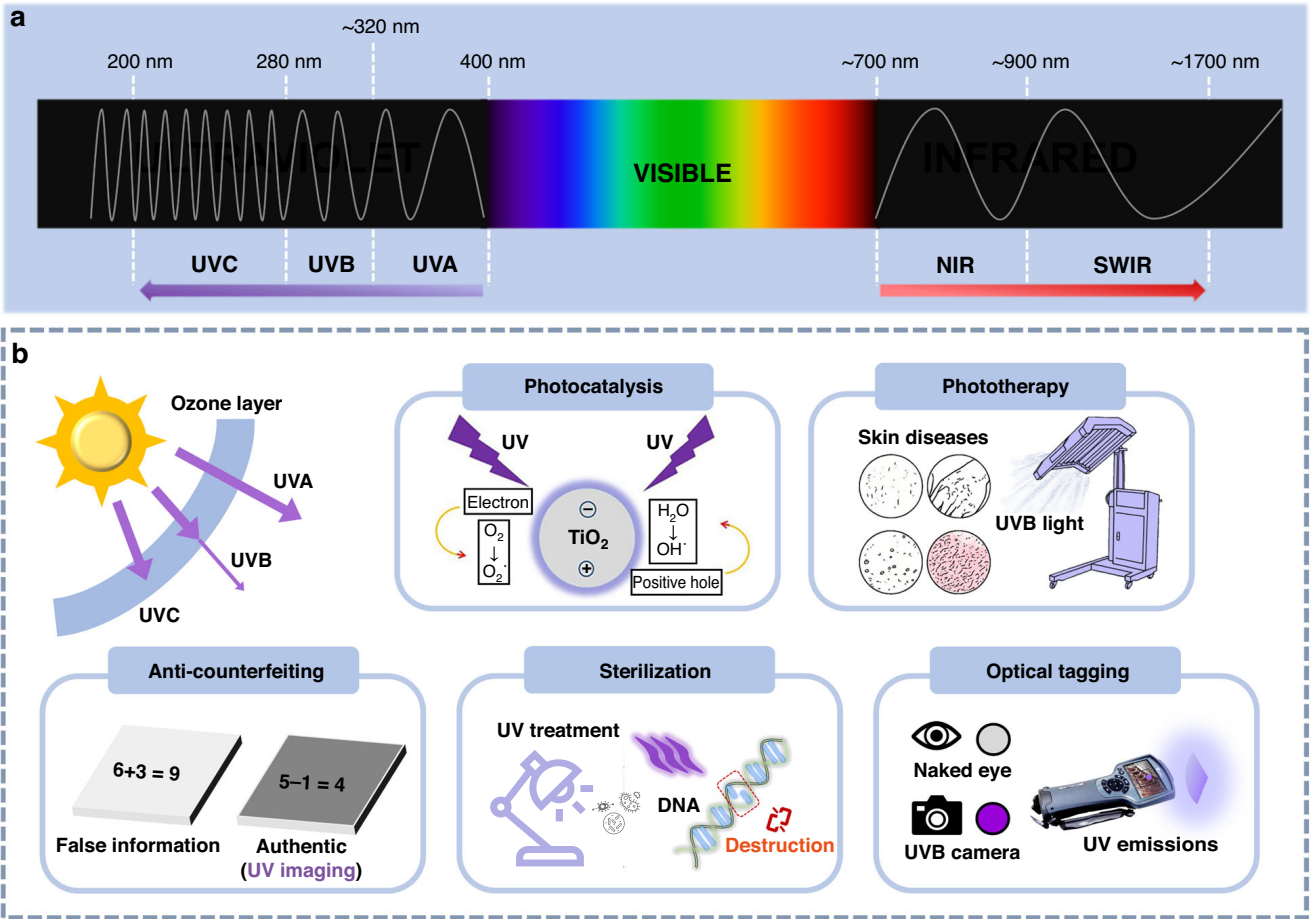
Classification and applications of UV light. **a** The part of the electromagnetic spectrum from UV to infrared wavelengths. **b** Potential applications of UVA, UVB, and UVC light for photocatalysis, phototherapy, anti-counterfeiting, sterilization, and optical tagging. Reproduced with permission from ref. ^8^. Copyright 2015 Elsevier

Ultraviolet (UV) light is high-energy electromagnetic radiation with wavelengths ranging from 10 to 400 nm^1,2^. Due to its significantly higher photon energy compared to visible light, UV radiation can induce unique electronic transitions in matter. UV radiation is typically categorized into four spectral regions based on wavelength: ultraviolet-A (UVA, 320–400 nm), ultraviolet-B (UVB, 280–320 nm), ultraviolet-C (UVC, 200–280 nm), and vacuum ultraviolet (VUV, 10–200 nm). Since only UV radiation above 200 nm can propagate through air, discussions on UV light and applications usually focus on the 200–400 nm range (Fig. 1a). The Sun serves as the Earth’s principal natural UV source, but approximately 97–99% of solar UV is absorbed by the ozone layer before reaching the Earth’s surface^3^. Of the remaining UV radiation, long-wave UVA dominates (95%) due to its superior atmospheric transmittance, while UVB accounts for only ~5%, with intensity varying based on meteorological conditions. UVC radiation is fully blocked by the ozone layer and fails to penetrate to the surface of the Earth^4^.

The wavelength-dependent properties of each UV band determine their respective functional advantages in practical applications (Fig. 1b)^5–7^. UVA processes excellent penetration capabilities, making it effective in turbid media such as wastewater and organic solvents. It also matches well with the band gaps of semiconductor photocatalysts such as TiO_2_, enabling widespread photocatalysis applications^8–10^. UVB radiation has shown therapeutic value, particularly in immune modulation^11^. Narrowband UVB (NB-UVB, 310–313 nm) has been clinically validated for treating skin disorders such as vitiligo and psoriasis^12^. High-energy UVC photons are highly effective for microbial inactivation, disrupting nucleic acids and thereby reducing microbial activity^13^. Emerging far-UVC (200–230 nm) technologies are advancing disinfection capabilities by inactivating pathogens while posing minimal risk to human skin, enabling use in occupied environments^14–16^. These broad-spectrum applications underscore the irreplaceable scientific and technological value of UV radiation in healthcare, industry, and environmental remediation.

**Fig. 2:**
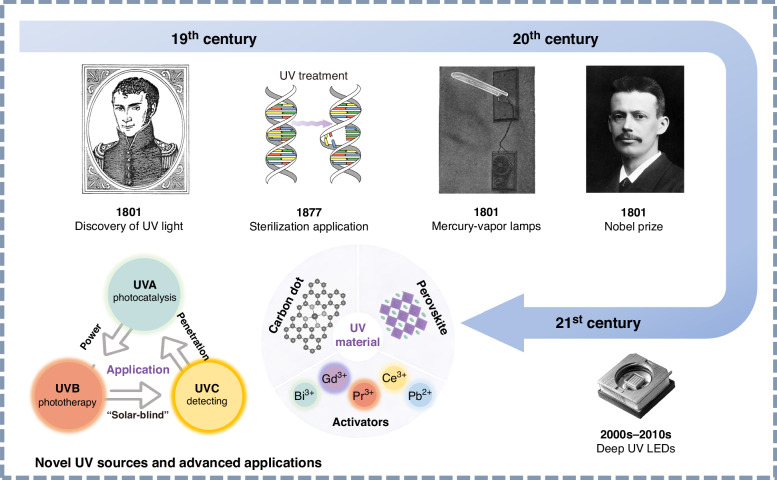
Development of UV light. The development history of UV light technology. Reproduced with permission from ref. ^33^. Copyright 2024 Elsevier

The evolution of UV technology from fundamental research to applied science has been marked by several milestones (Fig. 2). Since the discovery of UV light by Johann Wilhelm Ritter in 1801, its applications have expanded significantly^17^. In 1903, Niels Finsen, a Danish physician, was awarded the Nobel Prize in Physiology or Medicine for pioneering phototherapy using UV light^18^. The development of artificial UV sources—including mercury vapor lamps, excimer lasers, and UV LEDs^19–21^—has further enabled applications in sterilization, therapy, and photocatalysis^22,23^. More recently, novel UV-emitting materials such as metal-ion-doped UV luminescent materials, perovskite^24–27^, and carbon quantum dots^28–32^ have significantly enriched UV luminescent systems and driven technological innovation^33^. Among these, inorganic UV luminescent materials doped with rare-earth or heavy main-group metal ions have consistently played a central role due to their excellent spectral characteristics, high photostability, and tunable emission properties^34,35^.

Moreover, diverse UV luminescence modes in inorganic materials are being increasingly explored, extending the functional boundaries of UV light sources. For instance, inorganic UV persistent luminescence materials enable “self-luminescence” through energy storage and delayed emission^36^. UV upconversion phosphors enable the conversion of visible or infrared light into high-energy UV radiation through a nonlinear optical process^37^^,38^, while mechanoluminescent materials allow real-time conversion of mechanical energy into high-energy UV light emission^39,40^. These breakthroughs not only enhance the potential of inorganic UV phosphors but also provide new flexibility for engineering next-generation UV light sources. Therefore, a comprehensive review of rare-earth and heavy main-group metal ion-doped inorganic UV luminescent materials is both timely and necessary.

This review presents the development of these inorganic UV luminescent materials, covering fundamental luminescence mechanisms, recent research advances, and potential applications. We summarize significant advancements across UV persistent luminescence, upconversion luminescence, and mechanoluminescence systems, along with their functional characteristics and spectral properties. Finally, current challenges and future research directions are outlined to inspire continued progress in high-performance inorganic UV luminescent materials.

## Luminescence mechanisms of doped inorganic UV phosphors

**Fig. 3:**
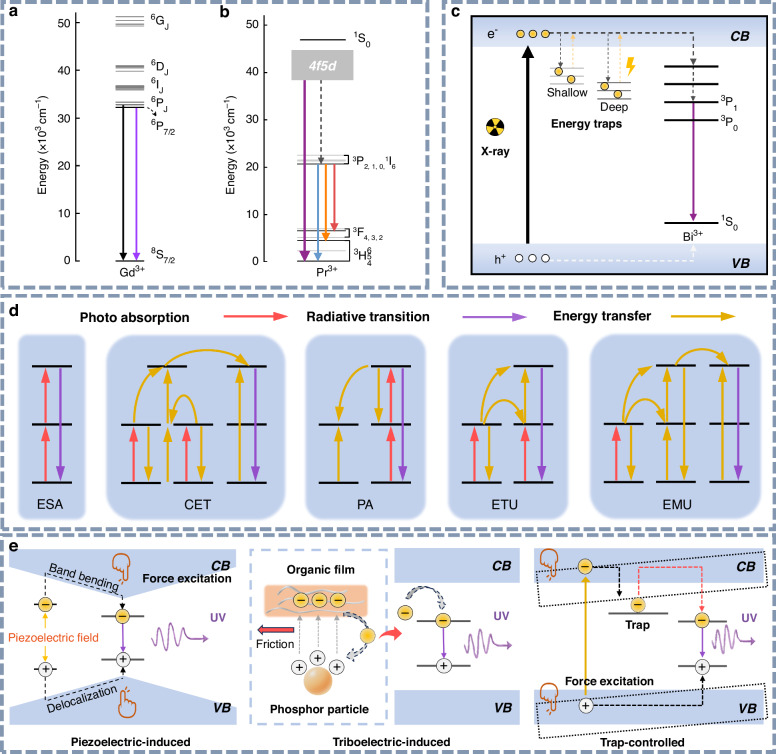
The mechanism of UV light emission. **a** Electronic energy-level diagram of Gd^3+^. **b** Electronic energy-level diagram of Pr^3+^. **c** Schematic representation of the UV afterglow mechanism in X-ray-excited Bi^3+^-doped persistent phosphors. **d** Schematic representation of upconversion luminescence mechanisms: ESA, CET, PA, ETU, and EMU. **e** Schematic illustration of the proposed mechanoluminescence mechanisms: piezoelectric-induced, triboelectric-induced, and trap-controlled models

Metal ions doped inorganic UV phosphors typically consist of a host material and a luminescent center. Inorganic materials exhibiting UV luminescence commonly utilize rare-earth ions (e.g., Pr^3+^, Gd^3+^, Ce^3+^, Tm^3+^, Er^3+^, Nd^3+^, and Tb^3+^) and heavy main-group metal ions (e.g., Bi^3+^ and Pb^2+^) as luminescent centers. Typical host materials include wide-bandgap fluorides, oxides, and oxygenated acid salts such as silicates, germanates, borates, and phosphates. Upon exposure to external excitation sources, such as short-wavelength UV light or X-rays, the excitation energy is first absorbed by the host matrix and subsequently transferred to the emitting center. This process promotes the ions from their ground state to an excited energy state. The excited ions subsequently undergo non-radiative energy loss through thermal or lattice vibrations, ultimately reaching a more stable excited state. Upon relaxation, electrons return to the ground state through radiative transitions, resulting in UV emission^41^. We present three typical UV transitions: the 4f→4f and 4f→5d transitions of rare-earth ions, as well as the ^3^P_1_→^1^S_0_ transition of heavy main-group metal ions (Fig. 3).

As depicted in Fig. 3a, Gd^3+^ is considered a favorable UV emitter due to its high-energy excited levels (^6^P_J_, ^6^I_J_, and ^6^D_J_), which are located above 32,000 cm^–1^. Transitions from ^6^P_J_ excited states to the ^8^S_7/2_ ground state of Gd^3+^ yield characteristic single-line UVB emissions^42,43^. These 4f→4f intra-configurational transitions are typically unaffected by the crystal field environment of the host material. However, the stable half-filled 4f^7^ electronic configuration of Gd^3+^ makes it resistant to oxidation or reduction in an inorganic matrix. To induce UV emission in Gd^3+^, the incorporation of sensitizer ions such as Bi^3+^, Pr^3+^, and Ce^3+^ ^44^, or the use of high-energy excitation sources, is often required^45^. Additionally, UV luminescence can also result from the 4f5d→4f transitions of rare-earth ions, resulting in broadband emissions whose wavelengths are influenced by the coordination environment. For example, the 4f5d→4f transitions of Pr^3+^ and Ce^3+^ ions are responsible for their high-efficiency broadband UV emissions^46^. As shown in Fig. 3b, Pr^3+^ processes a high-energy 4f5d state and can emit in the UV range when the excited electrons relax from the lowest 4f5d state to the ^3^H_4_ ground state^47^. To achieve effective UV emissions in Pr^3+^-doped phosphors, the Stokes shift associated with the 4f5d→4f transitions must not exceed ∼3000 cm^−1^ (0.37 eV), which places specific requirements on the choice of host materials^48^. For UV emissions involving heavy main-group metal ions, ns^2^ ions such as Bi^3+^ and Pb^2+^ play a pivotal role. The 6 s electrons in these ions exhibit strong sensitivity to the crystal field of the host lattice, and the position of their energy level can vary significantly across different host materials^49,50^. For example, the UV emission of Bi^3+^ is attributed to the ^3^P_1_→^1^S_0_ transition, where a weaker crystal field enhances the energy of this transition and induces a blueshift in the emission wavelength.

The luminescence mechanisms underlying afterglow materials have been extensively studied, with the prevailing models including the conduction band-valence band model, the quantum tunneling mechanism, and the oxygen vacancy mechanism. Among them, the conduction band-valence band model can be further subdivided into the hole model, the electron model, and the energy-band engineering model^51–53^. However, there remains no consensus regarding a specific model to fully explain the trapping and de-trapping processes of charge carriers in different types of long-afterglow materials. In this study, we use the widely accepted electron model to elucidate the luminescence mechanism of X-ray-excited Bi^3+^-doped UV persistent phosphors, as illustrated in Fig. 3c. Under X-ray excitation, electrons from the valence band (VB) of the host material are promoted to the conduction band (CB), generating holes in the VB. These excited electrons subsequently occupy energy traps, while the holes are captured by Bi^3+^ ions^54–56^. After ceasing X-ray irradiation, thermally induced vibrations can liberate electrons from both shallow and deep traps, allowing them to transition into the conduction band. These electrons then recombine with the ionized Bi^3+^ emitter, resulting in UV persistent luminescence. Even after prolonged decay, a substantial number of electrons remain trapped in deeper states. Upon exposure to ambient stimuli, such as heat or light, these electrons in deep traps are released into the CB and relax to the ground state via radiative transitions, thereby producing enhanced UV afterglow^57–60^.

Upconversion luminescence mechanisms are generally classified into five distinct types: excited-state absorption (ESA), energy transfer upconversion (ETU), cooperative energy transfer (CET), photon avalanche (PA), and energy migration-mediated upconversion (EMU)^61–63^. These luminescence mechanisms exhibit markedly different energy transfer pathways, each associated with varying upconversion efficiencies. As shown in Fig. 3d, the upconversion (UC) process involves the sequential absorption of two or more photons, promoting electrons from the ground state to higher energy levels, which then return to the ground state via radiative transitions, resulting in the emission of upconversion luminescence^64^. The ESA process requires exceptionally long lifetimes for intermediate energy levels, whereas the CET mechanism inherently lacks such intermediate states. As a result, both ESA and CET typically exhibit significantly lower upconversion efficiencies compared to other mechanisms. The PA mechanism is characterized by a distinct excitation threshold, below which upconversion luminescence is virtually undetectable. In contrast, ETU demonstrates superior efficiency due to the incorporation of sensitizer ions with simplified energy levels and enhanced photon absorption cross-sections. These sensitizers efficiently capture the excitation energy and relay it to the activators, consequently enhancing the overall upconversion efficiency. EMU, an extension of the ETU, also displays high upconversion efficiency. Consequently, ETU and EMU dominate in UV upconversion luminescence systems. It is noteworthy that most upconversion systems exhibit the coexistence of multiple mechanisms, rather than relying on a single pathway. While upconversion luminescence from infrared to visible light can be attributed to one or more of the five mechanisms mentioned above, the visible-to-UV upconversion has been reported exclusively for two mechanisms, ESA and ETU^65^.

The underlying mechanisms of mechanoluminescence remain incompletely understood because of the multi-step energy transfer processes involved. Currently, three principal mechanistic models are widely recognized (Fig. 3e). Piezoelectric-induced mechanoluminescence occurs in non-centrosymmetric or defect-engineered piezoelectric phosphors. Under applied stress, the resulting internal piezoelectric field lowers the energy barrier for detrapped electrons, enabling their recombination with luminescent centers to produce light emission^66^. In contrast, triboelectric-induced mechanoluminescence arises from interfacial triboelectrification during friction or deformation, which accounts for mechanoluminescence behavior in centrosymmetric systems lacking intrinsic piezoelectricity^67^. The third trap-controlled mechanoluminescence model relies on the storage of charge carriers within defect-related energy traps^68^. Mechanical excitation provides the energy required to release these trapped charge carriers, which subsequently migrate and recombine radiatively.

## Inorganic ultraviolet luminescent materials

### Inorganic UV persistent luminescence materials

**Fig. 4:**
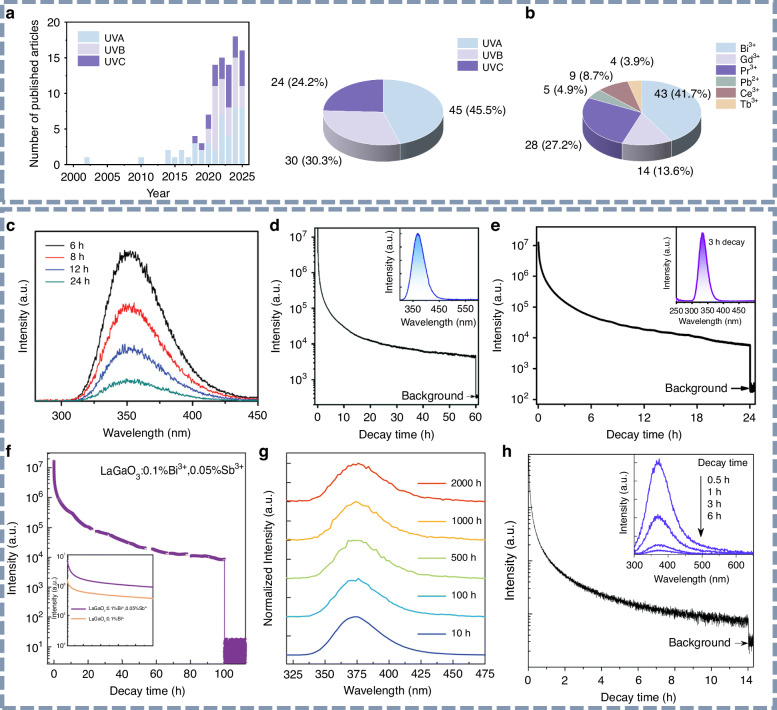
UVA persistent luminescence materials. **a** The number and proportion of publications relevant to UVA, UVB, and UVC persistent luminescence from 2000 to 2025. **b** The proportion of publications relevant to various UV emitters. **c** Persistent luminescence emission spectra of the LiYGeO_4_:Bi^3+^ phosphor at different decay instants. Reproduced with permission from ref. ^76^. Copyright 2019 John Wiley & Sons, Inc. **d** Decay curve of the LiScGeO_4_:Bi^3+^ persistent phosphor monitored at 365 nm. The upper inset is the emission spectrum recorded after 1 hour decay. Reproduced with permission from ref. ^77^. Copyright 2020 Royal Society of Chemistry. **e** Decay curve of the Sr_3_Sc_2_Ge_3_O_12_:Bi^3+^ persistent phosphor recorded at 333 nm. The upper inset is the afterglow emission spectrum recorded after 3 h decay. Reproduced with permission from ref. ^83^. Copyright 2022 Royal Society of Chemistry. **f** Decay curves and **g** corresponding afterglow emission spectra of the LaGaO_3_:Bi^3+^,Sb^3+^ phosphor at different decay instants. Reproduced with permission from ref. ^84^. Copyright 2024 Royal Society of Chemistry. **h** Decay curve of the Sr_2_MgGe_2_O_7_:Pb^2+^ persistent phosphor recorded at 370 nm. The upper inset is the afterglow emission spectrum. Reproduced with permission from ref. ^90^. Copyright 2016 Royal Society of Chemistry

As a unique class of energy-storage luminescent materials, persistent phosphors are capable of continuing to emit light for a certain period after the cessation of external excitation. This distinctive “self-sustained” luminescence property grants afterglow materials broad application prospects^69^. In particular, afterglow materials in the visible and infrared wavelength bands have garnered significant research interest, leading to important applications in fields such as night vision surveillance and bio-imaging. In contrast, research on UV persistent luminescence materials has been comparatively underdeveloped, primarily due to the limited exploration of material systems and energy-trap structures. However, recent advances in the design of large-bandgap host materials and the introduction of high-energy excitation sources have facilitated the development of persistent phosphors that emit in the UV spectrum, particularly in the deep-UV (200–320 nm) range. As shown in Fig. 4a, articles published between 2000 and 2025 on UV persistent luminescence have been categorized according to their emission wavelengths. It is evident that UVA afterglow materials have been studied since as early as 2003, accounting for the majority of subsequent reports. Among these, Bi^3+^-doped UVA persistent phosphors have been extensively investigated. More recently, research has shifted towards deep-UV afterglow materials, particularly UVB persistent luminescence systems featuring Gd^3+^ as an emitter, which have been widely reported, accounting for nearly half of the publications. Additionally, the exploration of UVC long afterglow materials has garnered increasing attention, with notable breakthroughs in both emission wavelength and afterglow intensity. Over this period, UV persistent luminescence has been achieved in a variety of luminescent centers, with heavy main-group metal ions, such as Bi^3+^, and rare earth ions, including Pr^3+^ and Gd^3+^, representing more than 80% of the reported studies (Fig. 4b). In this section, we summarize the development of UV persistent luminescence materials according to their emission wavelengths.

#### UVA persistent luminescence materials

The development of UV afterglow materials began with UVA persistent phosphors, which have undergone the most comprehensive exploration in terms of material systems and luminescence properties. Here, we provide a chronological summary of the reported UVA persistent luminescence materials, categorized by their respective emitters. The detailed properties of these materials are presented in Table 1.

**Table 1:** The detailed luminescence properties of UVA persistent phosphors

Emitter	Material system	Excitation peak (nm)	Emission peak (nm)	Persistent duration	Trap depth (eV)	Ref.
Bi^3+^	CdSiO_3_:Bi^3+^	221	360	/	/	2014^70^
	CdSiO_3_:Bi^3+^,Dy^3+^	233/274	360	/	/	2014^71^
	CdSiO_3_:Gd^3+^,Bi^3+^	254	344	6 h	0.54	2016^72^
	NaLuGeO_4_:Bi^3+^,Eu^3+^	254	400	>63 h	0.62	2017^73^
	LiYGeO_4_:Bi^3+^	254	350	>72 h	0.694, 0.806, 0.898	2019^76^
	LiScGeO_4_:Bi^3+^	254	365	>120 h	0.816, 1.042	2020^77^
	LiScGeO_4_:Bi^3+^	254	361	12 h	0.718, 0.900, 0.994, 1.746	2020^78^
	Sr_3_Y_2_Ge_3_O_12_:Bi^3+^	254/sunlight	354	60 h	0.726, 0.836, 1.200	2021^80^
	Sr_3_Sc_2_Ge_3_O_12_:Bi^3+^	254/sunlight	333	>12 h	0.89–1.12	2022^83^
	Mg_2_GeO_4_:Bi^3+^,Li^+^	254	350	>100 h	0.57–0.59, 0.81–0.90, 0.98–1.04	2023^74^
	BaSc_2_Ge_3_O_10_:Bi^3+^	254	370	>1.5 h	0.242, 0.451, 0.685	2023^81^
	LaGaO_3_:Bi^3+^,Sn^3+^	X-ray	372	2000 h	0.78, 1.12	2024^84^
	CaMgGeO_4_:Bi^3+^	254	346	24 h	0.678, 0.772	2024^75^
	Y_3_Ga_3_MgSiO_12_:Bi^3+^	254	324	>8 h	0.71–0.79	2024^82^
Pb^2+^	SrO:Pb^2+^	254	390	>1 h	/	2004^85^
	Sr_2_MgGe_2_O_7_:Pb^2+^	254	370	>12 h	0.726	2015^90^
Ce^3+^	CaB_2_O_4_:Ce^3+^	254	365	>15 h	0.52	2019^86^
	Ca_3_(PO_4_)_2_:Ce^3+^	254/X-ray	360	>16 h	0.75	2024^87^
Tb^3+^	Ca_3_Ga_2_Ge_3_O_12_:Tb^3+^,Tm^3+^	254	385	>1000 s	0.65, 0.82	2018^88^
	Cs_2_NaYF_6_:Tb^3+^	X-ray	380	>50 h	/	2020^89^

Owing to its broad and tunable UV emission in various host lattices, Bi^3+^ has been recognized as a favorable luminescent ion for UV-emitting applications. In recent years, the UV persistent luminescence of Bi^3+^-doped germanate, phosphate, and silicate systems has been extensively studied, with afterglow emissions predominantly in the UVA spectral region. In 2014, Yang et al. reported a Bi^3+^-doped UVA persistent phosphor, CdSiO_3_:Bi^3+^, which exhibited weak UVA afterglow at 360 nm^70,71^. Subsequently, the co-doping of Gd^3+^ ions significantly enhanced the UVA afterglow intensity, resulting in a persistent emission at 344 nm that lasted for over 6 h^72^. In 2017, Wang et al. developed a novel NaLuGeO_4_:Bi^3+^,Eu^3+^ phosphor, in which co-doping with Eu^3+^ introduced additional energy traps^73^. This work also proposed design strategies for UV persistent phosphors using this system as a model. Following this, a series of Bi^3+^-doped germanate phosphors was reported, all exhibiting UVA emissions with afterglow durations ranging from several hours to over tens of hours^74,75^. As illustrated in Fig. 4c, Shi et al. reported intense UVA afterglow in LiYGeO_4_:Bi^3+^ phosphor, with long-lasting afterglow at 350 nm for more than 72 h after 254 nm UV light irradiation^76^. Similarly, LiScGeO_4_:Bi^3+^ phosphor was developed to emit UVA afterglow centered at 365 nm, maintaining emission for over 120 h (Fig. 4d)^77,78^. This phosphor also exhibits strong photostimulated persistent luminescence (PSPL), allowing for the rejuvenation of long-decayed afterglow through short-time photostimulation^77^. Further studies demonstrated that the presence of energy traps around 0.7 eV in LiREGeO_4_ (RE = Y, Sc) structures makes them promising hosts for generating long-lasting UVA luminescence from Bi^3+^ ^79^.

In addition, garnet structures have been validated as excellent host materials for intense UVA afterglow, owing to their favorable crystal field environments and structural stability^80–82^. Liang’s group reported a Bi^3+^-doped Sr_3_Sc_2_Ge_3_O_12_ garnet-type persistent phosphor exhibiting UVA afterglow centered at 333 nm (Fig. 4e), which could be effectively charged using natural sunlight^83^. More recently, Liu et al. developed an X-ray-excited LaGaO_3_:Bi^3+^, Sb^3+^ phosphor with an oxygen-deficient perovskite structure, which demonstrated exceptional carrier-trapping capability^84^. As a result, this material produced ultra-long UVA afterglow lasting over 2000 h—representing the best-performing UVA persistent phosphor reported to date (Fig. 4f-g). Beyond Bi^3+^, other dopant ions such as Pb^2+^ ^85^, Ce^3+^ ^86,87^, and Tb^3+^ ^88,89^ have also been employed to achieve UVA persistent luminescence. For example, as illustrated in Fig. 4h, Liang et al. first reported a Pb^2+^-doped Sr_2_MgGe_2_O_7_ phosphor, which exhibited a broad-band afterglow covering the entire UVA spectral region^90^.

#### UVB persistent luminescence materials

**Fig. 5:**
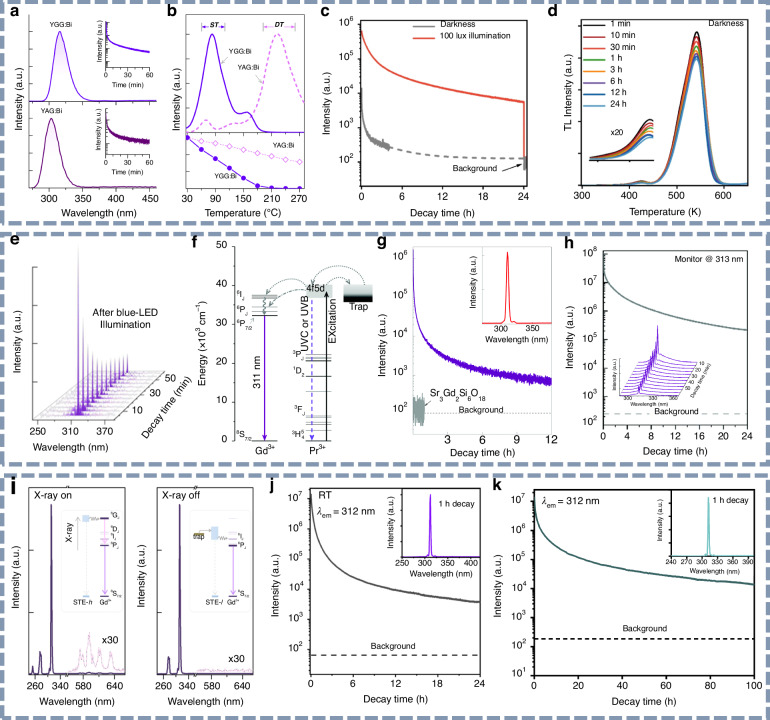
UVB persistent luminescence materials. **a** UV afterglow emission spectra of the Y_3_Ga_5_O_12_:Bi^3+^ and Y_3_Al_5_O_12_:Bi^3+^ phosphors after 30 min decay. The insets are persistent luminescence decay curves monitored at 316 and 303 nm, respectively. Reproduced with permission from ref. ^99^. Copyright 2020 Optica Publishing Group. **b** Thermoluminescence and photoluminescence thermal quenching of the Y_3_Ga_5_O_12_:Bi^3+^ and Y_3_Al_5_O_12_:Bi^3+^ phosphors. Reproduced with permission from ref. ^99^. Copyright 2020 Optica Publishing Group. **c** Persistent luminescence decay curves of the ScBO_3_:Bi^3+^ storage phosphor recorded at 299 nm in dark and bright (100 lux) environments. Reproduced with permission from ref. ^101^. Copyright 2024 Springer Nature. **d** Thermoluminescence spectra of the ScBO_3_:Bi^3+^ phosphor at different decay instants in darkness. Reproduced with permission from ref. ^101^. Copyright 2024 Springer Nature. **e** Upconverted persistent luminescence emission spectra of Lu_2_Al_2_Ga_3_O_12_:Pr^3+^,Gd^3+^ phosphor at different decay instants. Reproduced with permission from ref. ^102^. Copyright 2020 American Physical Society. **f** Schematic representation of persistent energy transfer from Pr^3+^ to Gd^3+^ in NB-UVB persistent phosphors. Reproduced with permission from ref. ^103^. Copyright 2021 Royal Society of Chemistry. **g** Decay curves of Sr_3_Gd_2_Si_6_O_18_:Pr^3+^ persistent phosphor and Sr_3_Gd_2_Si_6_O_18_ host monitored at 311 nm. The upper inset is the persistent luminescence emission spectrum after 10 min decay. Reproduced with permission from ref. ^103^. Copyright 2021 Royal Society of Chemistry. **h** Decay curve of the (Y, Gd)_3_Ga_5_O_12_:Bi^3+^ persistent phosphor monitored at 313 nm. The inset is the afterglow emission spectrum at different decay instants in the first 60 min. Reproduced with permission from ref. ^104^. Copyright 2021 Royal Society of Chemistry. **i** Steady-state photoluminescence emission and persistent luminescence emission spectra of the NaYF_4_:Gd^3+^ phosphor. The insets are the luminescence mechanisms. Reproduced with permission from ref. ^105^. Copyright 2025 John Wiley & Sons, Inc. **j** Persistent luminescence decay curve of the Sr_2_P_2_O_7_:Gd^3+^ phosphor monitored at 312 nm. The upper inset is the persistent luminescence emission spectrum after 1 h decay. Reproduced with permission from ref. ^109^. Copyright 2022 American Chemical Society. **k** Persistent luminescence decay curve of the LiCaPO_4_:Gd^3+^ phosphor monitored at 312 nm. The inset is the persistent luminescence emission spectrum after 1 h decay. Reproduced with permission from ref. ^110^. Copyright 2024 Royal Society of Chemistry

Since 2020, UVB persistent luminescence materials have been widely reported and are generally classified into two categories according to their emission characteristics: broadband and narrowband luminescence. In broadband UVB afterglow materials, Bi³⁺ and Pr³⁺ typically serve as the emitting centers^91–97^, while Ce³⁺ has also been reported in this context^98^. In contrast, Gd³⁺ is primarily responsible for narrowband UVB (NB-UVB) emissions. The detailed luminescence properties of these UVB persistent phosphors are summarized in Table 2. In 2020, Sun et al. reported UVB persistent luminescence in isostructural garnet phosphors, Y_3_Ga_5_O_12_:Bi^3+^ and Y_3_Al_5_O_12_:Bi^3+^, exhibiting afterglow peaks at 316 and 303 nm, respectively. These emissions remained detectable for over 1 hour following excitation with a 254 nm UV lamp (Fig. 5a)^99^. Notably, the authors highlighted discrepancies between thermoluminescence (TL) measurements and trap-related properties by comparing the thermal quenching behaviors of the two garnet hosts, offering valuable insights for subsequent TL analysis (Fig. 5b). Subsequently, Liang’s group achieved long-lasting UVB persistent luminescence in Y_3_Ga_5_O_12_:Bi^3+^ phosphors activated by natural sunlight, with an afterglow duration exceeding 60 hours^100^. More recently, Liang’s group reported a deep-trap UVB persistent phosphor, ScBO_3_:Bi^3+^, that exhibited a striking “glow-in-the-bright” property^101^. As illustrated in Fig. 5c, the persistent luminescence intensity of ScBO_3_:Bi^3+^ appears weak under dark conditions but is significantly intensified—by nearly two orders of magnitude—under continuous white LED illumination. Thermoluminescence analysis (Fig. 5d) reveals that a majority of electrons are retained in deep traps (>1.0 eV) even after prolonged decay, and the continuous stimulation by visible light promotes the rapid release of these deep-trapped carriers, thereby enhancing UVB afterglow intensity. This mechanism provides a promising strategy for the design of storage-type UV-emitting materials operable in bright environments.

**Table 2:** The detailed luminescence properties of UVB persistent phosphors

Emitter	Material system	Excitation peak (nm)	Emission peak (nm)	Persistent duration	Trap depth (eV)	Ref.
Bi^3+^	Y_3_Al_5_O_12_:Bi^3+^ Y_3_Ga_5_O_12_:Bi^3^	254	303	>1 h	/	2020^99^
	Y_3_Ga_5_O_12_:Bi^3+^	Sunlight/254	313	>60 h	0.67, 0.77, 0.84	2021^100^
	Mg_2_Lu_2_Ge_3_O_11_:Bi^3+^,Li^+^	254	310	>2 h	/	2022^91^
	LuY_2_AlGa_4_O_12_:Bi^3+^	254	310	>72 h	/	2022^92^
	Mg_8_Lu_2_Ge_6_O_23_:Bi^3+^	254	306	>12 h	0.66	2022^93^
	Ca_3_Ga_2_Ge_3_O_12_:Bi^3+^	254	315	>12 h	0.76, 0.81, 0.91, 0.96, 1.03	2024^94^
	ScBO_3_:Bi^3+^	X-ray	299	>24 h	1.29, 1.35	2024^101^
Pr^3+^	BaLu_2_Al_2_Ga_2_SiO_12_:Pr^3+^	254	301	>3 h	0.55–0.75	2021^95^
	Lu_3_Al_3_Ga_2_O_12_:Pr^3+^,Cr^3+^	254	302	60 h	0.67–1.01	2021^96^
	Y_3_Al_5_O_12_:Pr^3+^,Eu^3+^	X-ray	318	/	0.89, 1.63	2024^97^
Ce^3+^	SrAl_12_O_19_:Ce^3+^,Sc^3+^	254	300	>10 h	0.782, 0.866, 1.096	2024^98^
Gd^3+^	Lu_2_Al_2_Ga_3_O_12_:Pr^3+^,Gd^3+^	450	313	>1 h	0.696, 1.306	2020^102^
	Sr_3_Gd_2_Si_6_O_18_:Pr^3+^	254	311	>10 h	/	2021^103^
	Y_3_Ga_5_O_12_:Bi^3+^,Gd^3+^	254	313	>24 h	0.79	2021^104^
	LaMgAl_11_O_19_:Gd^3+^	X-ray	312	/	0.76	2021^105^
	Sr_2_P_2_O_7_:Gd^3+^	X-ray	312	>24 h	0.74, 0.95	2022^109^
	ScPO_4_:Gd^3+^	X-ray	313	>24 h	0.73, 0.89, 1.05	2023^107^
	CaF_2_:Gd^3+^	X-ray	313	>24 h	0.77, 0.89, 1.08	2024^108^
	LiCaPO_4_:Gd^3+^	X-ray	312	>100 h	/	2024^110^
	NaYF_4_:Gd^3+^	X-ray	311	/	/	2024^105^
	Sr_3_(PO_4_)_2_:Gd^3+^	X-ray	311, 313	>24 h	0.84	2025^111^

Regarding narrowband UVB (NB-UVB) luminescence, Gd^3+^ has emerged as a highly promising luminescent center. Due to its stable 4f^7^ electronic configuration, Gd^3+^ is inherently difficult to be excited directly with UV light. Therefore, various energy-transfer strategies have been developed to facilitate efficient NB-UVB emission. One common approach involves the introduction of sensitizer ions—such as Pr^3+^, Bi^3+^, and Pb^2+^—which possess broad absorption and emission bands. These ions effectively absorb UV light and can transfer the acquired energy to Gd^3+^. In 2020, Yan et al. first reported upconverted NB-UVB persistent luminescence in Lu_2_Al_2_Ga_3_O_12_:Pr^3+^,Gd^3+^ phosphor under blue LED excitation. In this system, Pr^3+^ absorbs two blue photons to populate energy traps, followed by continuous energy transfer to Gd^3+^, resulting in persistent line emission at 313 nm that lasted for approximately 1 h^102^. Subsequently, Wang et al. synthesized a series of Pr^3+^-, Pb^2+^- and Bi^3+^- sensitized NB-UVB persistent phosphors, showcasing efficient energy transfer to Gd^3+^. Among them, the representative Sr_3_Gd_2_Si_6_O_18_:Pr^3+^ phosphor exhibited NB-UVB afterglow centered at 311 nm, persisting for over 12 h following excitation by a 254 nm UV lamp (Fig. 5g)^103^. Building on similar design principles, Liang’s group further realized efficient NB-UVB persistent luminescence via energy transfer from Bi^3+^ to Gd^3+^ in the (Y, Gd)_3_Ga_5_O_12_:Bi^3+^ phosphor, as shown in Fig. 5h^104^. Notably, this work also demonstrated the feasibility of using natural sunlight as an effective excitation source, broadening the practical applicability of such materials.

Alternatively, the generation of self-trapped excitons under high-energy excitation sources, such as X-rays, can also enable efficient energy transfer to Gd^3+^, thereby facilitating NB-UVB persistent luminescence, as illustrated in Fig. 5i^105^. In 2021, Liu’s group first reported an X-ray-charged LaMgAl_11_O_19_:Gd^3+^ phosphor that exhibited ambient-stimulated NB-UVB light emission, thereby stimulating interest in exploring various environmental triggers for UV afterglow^106^. Subsequently, an increasing number of Gd^3+^-doped, large-bandgap host materials—such as phosphates and fluorides—have been developed to exhibit X-ray-excited NB-UVB persistent luminescence^107,108^. As shown in Fig. 5j, Liang’s group reported a novel NB-UVB persistent phosphor, Sr_2_P_2_O_7_:Gd^3+^, which demonstrated strong line emission at 312 nm lasting for >24 h following only a few minutes of X-ray irradiation^109^. Notably, ambient light stimulation was found to play a crucial role in enhancing the UVB emission. More recently, Liang’s group reported intense NB-UVB afterglow from a series of Gd^3+^-doped phosphate phosphors^110,111^, LiCaPO_4_:Gd^3+^ and Sr_3_(PO_4_)_2_:Gd^3+^. The former exhibited optimal emission intensity and an impressive afterglow exceeding 100 h (Fig. 5k), which is one of the UVB afterglow materials with the longest reported afterglow duration to date^110^. Through a robust combination of spectroscopic analysis and first-principles calculations, this study provides valuable insight into the nature of energy traps and the mechanisms governing afterglow in NB-UVB phosphors. The findings also offer a referential framework for future research on similar material systems.

#### UVC persistent luminescence materials

In the design of shorter-wavelength UVC persistent luminescence materials, Pr^3+^ and Bi^3+^ are commonly regarded as promising emitters due to their tunable high-energy emitting states. This is particularly true in wide-bandgap host materials with appropriate crystal field environments, where Pr^3+^ frequently enables effective UVC luminescence. In this section, we summarize recent advancements in UVC afterglow materials, with detailed information provided in Table 3.

**Fig. 6:**
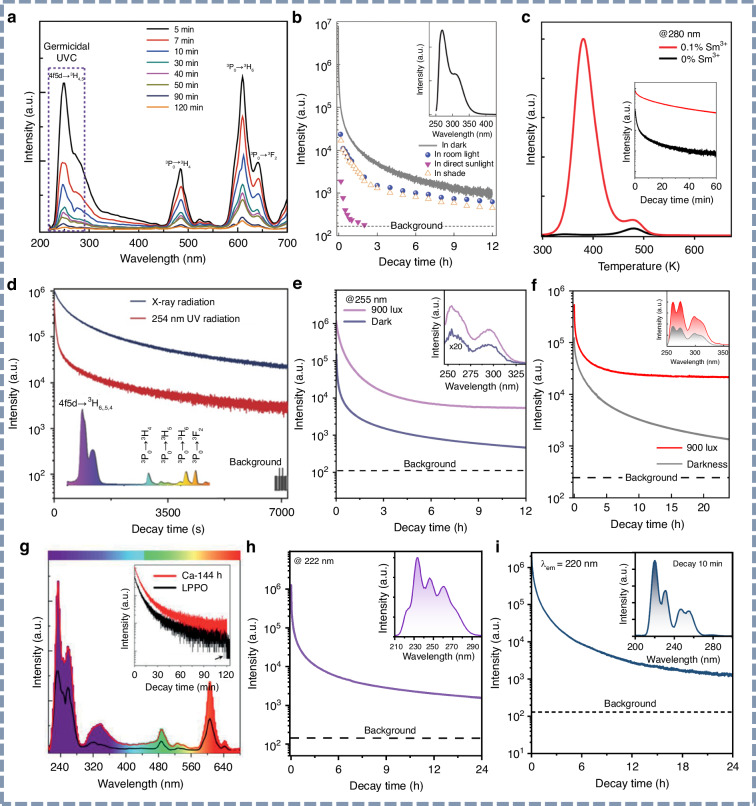
UVC persistent luminescence materials. **a** Emission spectra of the Cs_2_NaYF_6_:Pr^3+^ persistent phosphor at different afterglow times. Reproduced with permission from ref. ^112^. Copyright 2018 Springer Nature. **b** Persistent luminescence decay curves of the Ca_2_Al_2_SiO_7_:Pr^3+^ phosphor monitored at 268 nm. Reproduced with permission from ref. ^113^. Copyright 2020 Springer Nature. **c** Thermoluminescence spectra of the LiLuSiO_4_:Pr^3+^ and LiLuSiO_4_:Pr^3+^,Sm^3+^ phosphors. Inset is afterglow decay curves monitored at 280 nm. Reproduced with permission from ref. ^116^. Copyright 2022 Royal Society of Chemistry. **d** Afterglow decay curves of the Li_2_CaGeO_4_:Pr^3+^ phosphor after X-rays and 254 nm UV lamp irradiation. Reproduced with permission from ref. ^117^. Copyright 2021 Springer Nature. **e** Afterglow decay curves of the Li_2_CaSiO_4_:Pr^3+^ phosphor monitored at 255 nm in dark and bright (900 lux) environments. The upper inset is the afterglow emission spectrum recorded after 1 h decay. Reproduced with permission from ref. ^118^. Copyright 2023 Elsevier. **f** Persistent luminescence decay curves of the YBO_3_:Pr^3+^ phosphor monitored at 274 nm in dark and bright (900 lux) environments. The upper inset is the afterglow emission spectrum recorded after 30 min decay. Reproduced with permission from ref. ^119^. Copyright 2023 Royal Society of Chemistry. **g** Afterglow emission spectra of the LaPO_4_:Pr^3+^ phosphor after 5 min decay. The upper inset is the afterglow decay curves monitored at 231 nm. Reproduced with permission from ref. ^121^. Copyright 2020 John Wiley & Sons, Inc. **h** Afterglow decay curve of the Sr_2_P_2_O_7_:Pr^3+^ phosphor monitored at 222 nm. The upper inset is the afterglow emission spectrum. Reproduced with permission from ref. ^125^. Copyright 2023 Royal Society of Chemistry. **i** Afterglow decay curve of the CaSO_4_:Pr^3+^ phosphor monitored at 220 nm. The upper inset is the afterglow emission spectrum after 10 min decay. Reproduced with permission from ref. ^126^. Copyright 2025 Royal Society of Chemistry

In 2018, Sun and coworkers reported UVC persistent luminescence in X-ray-charged Cs_2_NaYF_6_:Pr^3+^ phosphor, featuring an emission peak at 250 nm and an afterglow duration exceeding 2 h (Fig. 6a)^112^. Subsequently, Pan’s group developed a series of Pr^3+^-doped silicate phosphors capable of emitting UVC afterglow (265–270 nm) for several hours following excitation by a relatively “low-energy” 254 nm UV lamp^113^. As shown in Fig. 6b, a representative Ca_2_Al_2_SiO_7_:Pr^3+^ phosphor demonstrated persistent UVC luminescence peaking at 268 nm for more than 12 h. Notably, the authors quantified the afterglow intensity at a decay time of 1 s, measuring a power density of 10.9 mW m⁻², thus offering a scientific approach for evaluating practical UVC afterglow performance. In addition, Wang et al. achieved dual-band UVC and UVB persistent luminescence by engineering the garnet structure, with emission maxima at 266 and 311 nm following excitation by a 254 nm UV lamp^114^. Liang’s group also reported several Pr^3+^-doped UVC persistent phosphors^115,116^. They employed the Vacuum Referred Binding Energy (VRBE) diagram as a strategic tool to enhance UVC afterglow performance in the LiLuSiO_4_:Pr^3+^ system. Specifically, they demonstrated that co-doping with trace amounts of Sm^3+^ could efficiently introduce a substantial number of electron traps, thereby significantly improving UVC persistent luminescence (Fig. 6c).

**Table 3:** The detailed luminescence properties of UVC persistent phosphors

Emitter	Material system	Excitation peak (nm)	Emission peak (nm)	Persistent duration	Trap depth (eV)	Ref.
Bi^3+^	YPO_4_:Bi^3+^	X-ray	240	>2 h	0.44, 0.88, 0.92	2021^122^
	LuPO_4_:Bi^3+^	X-ray	236	>12 h	0.68, 0.8	2022^123^
Pr^3+^	Cs_2_NaYF_6_:Pr^3+^	X-ray	250	>2 h	0.69, 0.83, 1.02	2018^112^
	LaPO_4_:Pr^3+^	X-ray	231	>2 h	/	2020^121^
	Ca_2_Al_2_SiO_7_:Pr^3+^	254	268	>12 h	0.776–0.988	2020^113^
	Lu_2_SiO_5_:Pr^3+^	254	270	>12 h	0.72, 0.82, 0.94, 1.08	2021^115^
	Li_2_CaGeO_4_:Pr^3+^	254/X-ray	245	>2 h	0.77–0.84, 0.96–1.06	2021^117^
	(Ca_1.5_Y_1.5_)(Al_3.5_Si_1.5_)O_12_:Pr^3+^	254	266/311	>12 h	0.42–0.70, 0.52–0.98	2022^114^
	LuPO_4_:Pr^3+^	X-ray	233	/	0.776, 0.976, 1.286	2022^124^
	LiLuSiO_4_:Pr^3+^,Sm^3+^	254/X-ray	270	>24 h	1.00–1.15	2022^116^
	YBO_3_:Pr^3+^	X-ray	274	>24 h	1.10–1.28	2023^119^
	Sr_2_P_2_O_7_:Pr^3+^	X-ray	222	>24 h	0.85–0.97, 1.18–1.43	2023^125^
	Li_2_CaSiO_4_:Pr^3+^	X-ray	255	>12 h	0.98–1.37	2023^118^
	Sr_3_(PO_4_)_2_:Pr^3+^	X-ray	268	>5 h	0.901, 0.947, 1.021	2025^120^
	CaSO_4_:Pr^3+^	X-ray	220	>24 h	0.76–1.07	2025^126^
Pb^2+^	CaSO_4_:Pb^2+^	X-ray	230	>24 h	0.86–1.14	2025^126^

In addition, a multi-responsive UVC persistent phosphor, Li_2_CaGeO_4_:Pr^3+^, was reported by Xia’s group, which can produce UVC emission under various excitation sources, including X-rays, 254 nm UV lamp, and blue lasers, as illustrated in Fig. 6d^117^. This multi-excitation capability offers new insights into the development of versatile and efficient UVC light sources. Similarly, Liang’s group achieved multi-responsive UVC luminescence in a related silicate system^118^. Upon excitation by X-rays or 450 nm blue LEDs/lasers, the Li_2_CaSiO_4_:Pr^3+^ phosphor exhibited intense UVC persistent luminescence or UVC upconversion emission. The UVC afterglow monitored at 255 nm persisted for over 12 h, and showed a marked enhancement under continuous white LED stimulation (Fig. 6e). In another study, Lv et al. systematically investigated the influence of ambient illumination on UVC persistent luminescence utilizing a deep-trap YBO_3_:Pr^3+^ phosphor^119^. As illustrated in Fig. 6f, the UVC afterglow intensity under white LED stimulation exceeded that in darkness by more than an order of magnitude after prolonged decay (>24 h). This remarkable "glow-in-the-daylight" phenomenon provides a strong foundation for developing UVC phosphors suitable for bright environments.

Beyond tuning UVC afterglow performance through environmental stimulation^120^, pushing the boundaries of luminescence performance indicators—such as achieving shorter emission wavelengths and higher afterglow intensity—represents another critical direction in UVC phosphor research. As illustrated in Fig. 6g, Sun’s group reported an X-ray excited LaPO_4_:Pr^3+^ phosphor with an afterglow emission peak at 231 nm and a persistent luminescence duration of up to 2 h^121^. Subsequently, UVC persistent luminescence with emission wavelengths below 250 nm was realized in Bi^3+^/Pr^3+^ doped phosphate systems, such as REPO_4_:Pr^3+^/Bi^3+^(RE = La, Lu)^122–124^. However, these materials generally exhibited relatively low UVC afterglow intensity. Recently, Liang’s group has developed a series of novel UVC persistent phosphors that effectively balance emission wavelength and afterglow intensity, thereby extending the emission range of UV phosphors into the far-UVC region (200–230 nm). As shown in Fig. 6h, Sr_2_P_2_O_7_:Pr^3+^ exhibited strong far-UVC afterglow centered at 222 nm, with a persistence duration exceeding 24 h and a record emission intensity of 35.42 mW m^−2^ at 30 s—representing the highest UVC afterglo intensity reported to date^125^. However, the central emission peak of this material lies at 233 nm, and achieving a higher proportion of emission within the far-UVC region remains desirable. To further address this, Liang’s group has recently developed two sulfate-based phosphors, CaSO_4_:Pr^3+^ and CaSO_4_:Pb^2+^, that push the emission wavelength even shorter^126^. In particular, CaSO_4_:Pr^3+^ exhibits a main afterglow emission peak at 220 nm—the shortest UVC afterglow wavelength reported to date, to the best of our knowledge. As illustrated in Fig. 6i, the far-UVC afterglow at 220 nm maintains a duration exceeding 24 h, highlighting its significant potential for far-UVC applications.

#### Upconversion charging UV persistent phosphors

In the development of UV persistent luminescence materials, high-energy excitation sources—such as X-rays and deep-UV light—are typically required to charge the phosphors. However, this requirement significantly limits their practical applications. As a result, the effective excitation of UV persistent phosphors using low-energy light has become a prominent research focus in the field. Interestingly, in certain specialized persistent luminescence systems, energy traps can be filled via the absorption of non-linear visible photons, leading to UV afterglow emission. This upconversion charging mechanism uses low-energy light as the excitation source. By employing a two-step dissociation excitation strategy, it allows effective trap filling without relying on high-energy irradiation, thereby offering a novel route for the functional activation of UV afterglow materials.

**Fig. 7:**
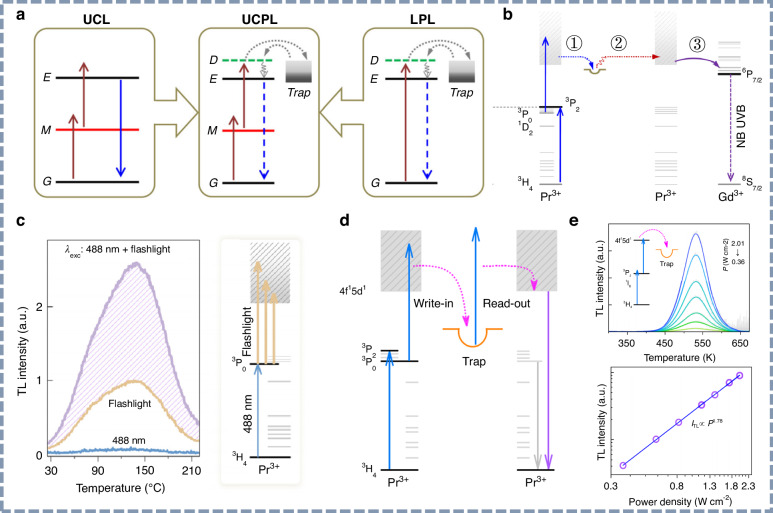
Upconversion charging UV persistent luminescence materials. **a** Schematic diagrams of upconversion luminescence, up-converted persistent luminescence, and persistent luminescence. Reproduced with permission from ref. ^127^. Copyright 2014 American Physical Society. **b** Schematic diagrams of up-converted NB-UVB persistent luminescence upon blue light illumination. Reproduced with permission from ref. ^101^. Copyright 2020 American Physical Society. **c** UCC performance of the Y_3_Al_2_Ga_3_O_12_:Pr^3+^ phosphor upon various excitation sources. Reproduced with permission from ref. ^132^. Copyright 2022 AIP Publishing. **d** Schematic diagrams of UCC for information storage and photostimulated luminescence for readout using a single-wavelength blue light. Reproduced with permission from ref. ^133^. Copyright 2023 John Wiley & Sons, Inc. **e** Verification of two blue-photon UCC process in the Y_3_Al_2_Ga_3_O_12_:Pr^3+^,Eu^3+^ phosphor. Reproduced with permission from ref. ^133^. Copyright 2023 John Wiley & Sons, Inc

In 2014, Pan’s group first proposed the concept of up-converted persistent luminescence by integrating the Yb^3+^/Er^3+^ ion pair with the NIR persistent phosphor Zn_3_Ga_2_GeO_8_:Cr^3+^, as illustrated in Fig. 7a^127^. This approach enabled near-infrared persistent luminescence from Cr^3+^ at 700 nm upon low-energy excitation using a 980 nm laser. Subsequently, Chen et al. proposed the innovative two-photon up-conversion charging (UCC) mechanism as a means of low-energy excitation in NIR persistent phosphors^128^. Since then, the use of low-energy light as an excitation source has been thoroughly validated and increasingly applied across various infrared afterglow material systems^129–131^.

In 2020, Liu’s group initiated the application of upconversion charging (UCC) in UV-emitting persistent phosphors^101^. As illustrated in Fig. 7b, the mechanism of up-converted NB-UVB persistent luminescence is clearly elucidated. Under continuous blue-light illumination, Pr^3+^ ions are excited to the 4f5d state via a two-photon absorption process, allowing the resulting excited electrons to be captured by energy traps. Upon cessation of external excitation, the trapped electrons gradually recombine with the ionized Pr^3+^ ions, generating UV persistent luminescence. Simultaneously, energy transfer from Pr^3+^ to Gd^3+^ gives rise to the observed NB-UVB afterglow. To date, most reported UCC technologies have relied on monochromatic excitation sources. However, extending this approach to polychromatic light sources offers additional potential for practical applications. Shi et al. demonstrated the feasibility of using a flashlight as an excitation source to generate UV persistent luminescence from Y_3_Al_2_Ga_3_O_12_:Pr^3+^ phosphor^132^. As shown in Fig. 7c, the thermoluminescence spectra revealed significant differences under various excitation sources, and a notable enhancement in thermoluminescence intensity was observed under combined excitation. These results support an excited-state absorption mechanism during the UCC process. Building upon this strategy, Liao et al. utilized single-wavelength low-energy light to simultaneously achieve both the trap-filling and de-trapping processes in the Y_3_Al_2_Ga_3_O_12_:Pr^3+^,Eu^3+^ UV persistent phosphor^133^. As shown in Fig. 7d, this single-wavelength write-read scheme significantly simplifies information storage and retrieval compared to conventional afterglow systems. Moreover, thermoluminescence measurements (Fig. 7e) further confirm that the UV UCC mechanism originates from a two-photon absorption process.

### UV upconversion luminescence materials

UV upconversion luminescence materials can transform low-energy near-infrared (NIR) or visible photons into high-energy UV emission. These materials exhibit characteristics of low-energy photon absorption and high-energy photon emission, enabling practical applications across various fields, including solid-state lasers, sterilization and disinfection, and photomedicine. Over the past two decades, researchers have gained a deeper understanding of the physical processes underlying energy transfer in upconversion luminescence. Efficient UV upconversion luminescence has been achieved in a range of material structures, including crystals, nanocrystals, and ceramics, through various technological approaches such as core-shell nanostructure design and the use of multi-wavelength excitation sources. In this paper, we summarize key advancements in the development of inorganic UV upconversion luminescence materials, categorizing them into two-photon and multiphoton upconversion luminescence, based on a chronological framework.

#### Two-photon absorption UV upconversion luminescence materials

**Fig. 8:**
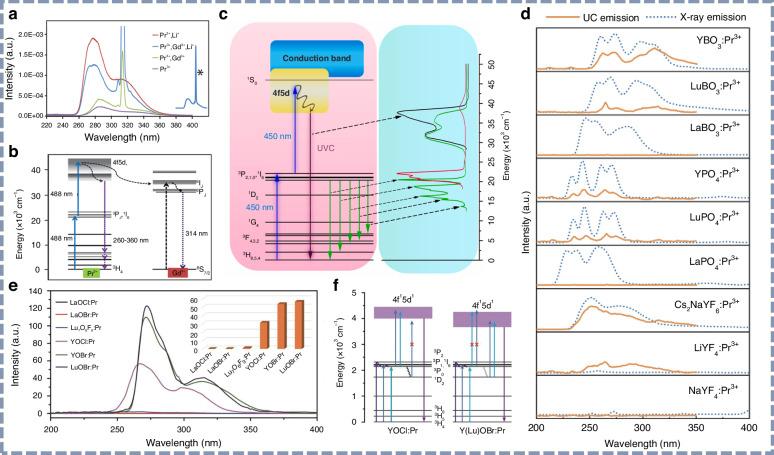
Two-photon absorption UV upconversion luminescence. **a** UVC upconversion emission spectra and **b** energy level diagram of Y_2_SiO_5_:Pr^3+^,Gd^3+^,Li^+^ phosphors upon 488 nm light excitation. Reproduced with permission from ref. ^137^. Copyright 2011 American Chemical Society. **c** Energy level diagram and upconversion luminescence spectra of the Li_2_SrSiO_4_:Pr^3+^ phosphor. Reproduced with permission from ref. ^142^. Copyright 2020 Elsevier. **d** UV upconversion luminescence and radioluminescence spectra of Pr^3+^-doped inorganic phosphors. Reproduced with permission from ref. ^147^. Copyright 2022 Optica Publishing Group. **e** UV upconversion luminescence spectra of REOX:Pr^3+^(RE = La, Y, or Lu; X = Cl, or Br) upon 450 nm light excitation. Reproduced with permission from ref. ^148^. Copyright 2024 John Wiley & Sons, Inc. **f** Energy level diagrams of YOCl:Pr^3+^ and Y(Lu)OBr:Pr^3+^ phosphors. Reproduced with permission from ref. ^148^. Copyright 2024 John Wiley & Sons, Inc

Pr^3+^ is recognized as a favorable UV upconversion emitter due to its characteristic and versatile energy-level configuration. Upon blue excitation, Pr^3+^ ions in upconversion phosphors undergo a two-photon absorption process, where electrons transition from the ^3^H_4_ ground level to intermediate ^3^P_J_ states, followed by excitation into the 4f5d manifold, leading to UV photon emission^134^. The detailed luminescence properties of the two-photon absorption UV upconversion phosphors are summarized in Table 4. Since 2006, the single-crystal Y_2_SiO_5_:Pr^3+^ has been identified as a potential candidate, with Jiang’s group using 488 and 532 nm lasers to generate UV upconversion luminescence^135,136^. Subsequently, Kim’s research group advanced the Y_2_SiO_5_:Pr^3+^ upconversion phosphor by introducing flux additives and utilizing polychromatic excitation^137–140^. As shown in Fig. 8a, Li-codoping has been shown to enhance UVC upconversion luminescence by modifying both the local activator centers and the surrounding phosphor lattice. Moreover, surface coatings and ceramics of synthesized Y_2_SiO_5_:Pr^3+^ have been demonstrated to effectively inhibit and sterilize bacteria. Following these developments, Yang and coworkers reported several UVC upconversion phosphors in Pr^3+^-doped inorganic materials^141–146^. As shown in Fig. 8c, the Li_2_SrSiO_4_:Pr^3+^ phosphor is capable of converting 450 nm blue light into UVC light through a two-photon upconversion process. For the entire UV emission range, the power density of this phosphor can reach 0.25 mW/cm^2^ (600 mW excitation). The researchers have also explored UV upconversion luminescence in other material systems, including silicates, phosphates, borates, and fluorides (Fig. 8d), and verified the feasibility of using sunlight as an excitation source^147^. Recently, Du et al. developed a series of Pr^3+^-doped rare-earth oxyhalides (YOCl, YOBr, and LuOBr), achieving efficient UV upconversion luminescence (Fig. 8e) and providing new insights into the host effects on upconversion processes^148^. By tailoring the oxyhalide host composition through crystal compositional engineering, the 4f5d energy level of Pr^3+^ can be effectively modulated. As shown in Fig. 8f, host composition modifications optimize absorption in intermediate states and suppress non-radiative relaxation, leading to enhanced UV upconversion.

**Table 4:** The detailed luminescence properties of the two-photon absorption UV upconversion phosphors

Material system	Emitter	Sensitizer	Excitation peak (nm)	Emission peak/band (nm)	Ref.
Y_2_SiO_5_:Pr^3+^	Pr^3+^	/	488	260–350	2006^135^
Y_2_SiO_5_:Pr^3+^	Pr^3+^	/	532	265–350	2007^136^
Y_2_SiO_5_:Pr^3+^,Gd^3+^,Li^+^	Pr^3+^	/	488	260–380	2011^137^
Y_2_SiO_5_:Pr^3+^,Li^+^	Pr^3+^	/	488	260–360	2012^138^
Y_2_SiO_5_:Pr^3+^,Li^+^	Pr^3+^	/	488 + 515/589	277	2013^139^
Y_2_SiO_5_:Pr^3+^,Li^+^	Pr^3+^	/	447	278	2014^140^
LiYF_4_:Pr^3+^	Pr^3+^	/	488/sunlight	/	2016^141^
Li_2_SrSiO_4_:Pr^3+^	Pr^3+^	/	450	240–350	2021^142^
YPO_4_:Pr^3+^	Pr^3+^	/	450	234, 245, 262, 272	2022^147^
Ca_2_SiO_4_:Pr^3+^	Pr^3+^	/	450	275	2022^143^
Sr_2_SiO_4_:Pr^3+^	Pr^3+^	/	450	265	2022^144^
SrSiO_3_:Pr^3+^	Pr^3+^	/	450/sunlight	245–365	2023^145^
Li_2_SrGeO_4_:Pr^3+^	Pr^3+^	/	450/sunlight	260–360	2023^146^
YOCl, YOBr, LuOBr:Pr^3+^	Pr^3+^	/	450	250–350	2024^148^
YAl_3_(BO_3_)_4_:Pr^3+^,Gd^3+^	Gd^3+^	Pr^3+^	450	311	2021^83^
LiYSiO_4_:Pr^3+^,Gd^3+^	Gd^3+^	Pr^3+^	450	313	2022^150^
LiYO_2_:Ho^3+^,Gd^3+^	Gd^3+^	Ho^3+^	445	314	2023^151^

**Fig. 9:**
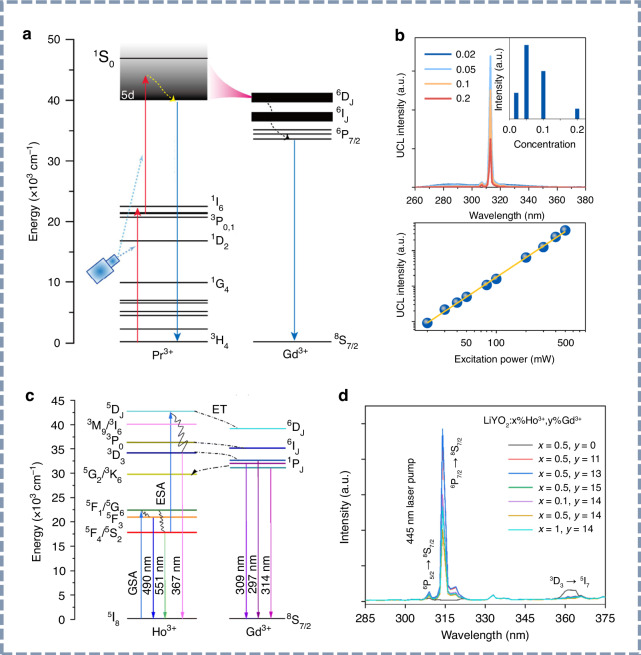
Gd^3+^-doped UVB upconversion luminescence. **a** Energy level diagram of Pr^3+^→Gd^3+^ energy transfer process. Reproduced with permission from ref. ^149^. Copyright 2026 American Chemical Society. **b** UVB upconversion luminescence spectra of the Li(Y,Gd)SiO_4_:Pr^3+^ phosphor upon 450 nm light excitation. Reproduced with permission from ref. ^150^. **c** Energy level diagram and **d** UVB upconversion luminescence spectra of LiYO_2_:Ho^3+^,Gd^3+^ phosphors upon 445 nm light excitation. Reproduced with permission from ref. ^151^. Copyright 2022 Royal Society of Chemistry

Additionally, by doping Gd^3+^ into Pr^3+^-doped systems, NB-UVB upconversion luminescence has been realized through Pr^3+^→Gd^3+^ energy transfer (Fig. 9a)^149^. As shown in Fig. 9b, Liang et al. reported the Pr^3+^-Gd^3+^ co-doped LiYSiO_4_ phosphor, which emits intense UVB radiation at 313 nm upon 450 nm blue light excitation^150^. The linear dependence of UV upconversion intensity on excitation power, observed in a double-logarithmic plot, indicates a two-photon mechanism. Zhao et al. developed the Ho^3+^-Gd^3+^ co-doped UVB upconversion phosphor, demonstrating enhanced blue-to-UVB conversion performance for use in optical temperature sensing (Fig. 9c, d)^151^.

#### Multiphoton absorption UV upconversion luminescence materials

The development and research of multiphoton UV upconversion luminescence materials primarily focus on achieving UV upconversion light emission via high-energy-density NIR laser excitation, with particular emphasis on the design and optimization of the energy transfer pathways. Notably, rare-earth fluorides are widely employed as host lattices for upconversion processes owing to their minimal phonon energies and outstanding chemical stability. The following section provides a chronological overview, with detailed luminescence properties summarized in Table 5.

**Fig. 10:**
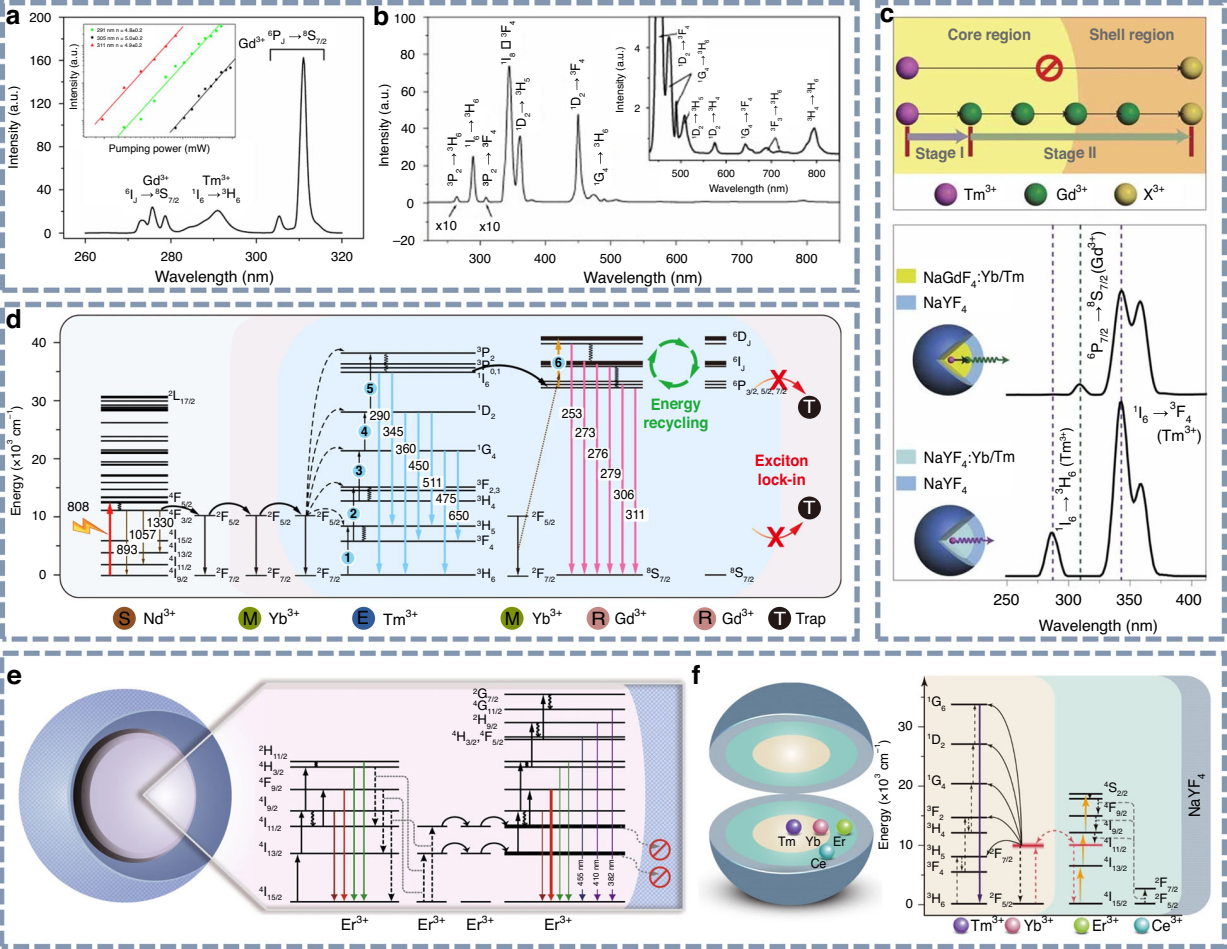
Multiphoton absorption UV upconversion luminescence. **a** UV upconversion emission spectrum of the YF_3_:Gd^3+^,Yb^3+^,Tm^3+^ nanocrystals. The inset is the pump-power dependence of the emissions. Reproduced with permission from ref. ^152^. Copyright 2008 Optica Publishing Group. **b** UV upconversion luminescence spectrum of NaYF_4_:Yb^3+^,Tm^3+^ microcrystals upon 980 nm laser excitation. Reproduced with permission from ref. ^156^. Copyright 2008 Optica Publishing Group. **c** Illustration of energy transfer mediated by the Gd sublattice across the core–shell interface from Tm^3+^ to activator ions, accompanied by the corresponding UV upconversion spectra. Reproduced with permission from ref. ^160^. Copyright 2011 Springer Nature. **d** Schematic energy-level diagram of the NaGdF_4_:Yb^3+^,Tm^3+^@NaGdF_4_:Yb^3+^@NaGdF_4_:Yb^3+^,Nd^3+^@NaGdF_4_ core-shell nanoparticle and the cascade energy transfer process. Reproduced with permission from ref. ^163^. Copyright 2021 Springer Nature. **e** Schematic of the energy-level structure and energy transfer pathway in the NaErF_4_@NaYF_4_ core-shell nanostructure. Reproduced with permission from ref. ^165^. Copyright 2019 Springer Nature. **f** Schematic of the energy-level structure and energy transfer pathway in the NaYF_4_:Yb^3+^/Tm^3+^@NaErF_4_:Ce^3+^@NaYF_4_ core-shell-shell structure. Reproduced with permission from ref. ^166^. Copyright 2022 Springer Nature

Qin’s group has made significant contributions to high-order multiphoton UV upconversion processes in fluoride hosts^152–154^. As shown in Fig. 10a, they reported the YF_3_:Gd^3+^,Yb^3+^,Tm^3+^ nanocrystals, which produce UV emission from Gd^3+^ ions when excited by a 980 nm laser. In this process, Yb^3+^ ions initially absorb the infrared photons and subsequently transfer the energy to Tm^3+^ ions. The energy is then further transferred to Gd^3+^ ions, resulting in UV emission. Furthermore, through a seven-photon upconversion luminescence process, the YF_3_:Gd^3+^,Yb^3+^,Tm^3+^ nanocrystals can generate vacuum ultraviolet (VUV) emissions under NIR laser excitation^155^. Besides, intense UV upconversion luminescence was observed in hexagonal NaYF_4_:Yb^3+^,Tm^3+^, where the unusual ^3^P_2_→^3^H_6_,^3^F_4_ transition of Tm^3+^ leads to UV emissions at 264 and 309 nm (Fig. 10b)^156^.

**Table 5:** The detailed luminescence properties of the multiphoton absorption UV upconversion phosphors

Material system	Emitter	Sensitizer	Excitation peak (nm)	Emission peak/band (nm)	Ref.
YF_3_:Gd^3+^,Yb^3+^,Tm^3+^	Gd^3+^	Yb^3+^,Tm^3+^	980	270–320	2008^152^
GdF_3_:Yb^3+^,Tm^3+^	Gd^3+^	Yb^3+^,Tm^3+^	980	246.2, 252.8	2008^153^
YF_3_:Gd^3+^,Yb^3+^,Tm^3+^	Gd^3+^	Yb^3+^,Tm^3+^	980	190–210	2015^155^
CaF_2_:Yb^3+^, Gd^3+^	Gd^3+^	Yb^3+^	980	314.8	2018^154^
NaYF_4_:Yb^3+^,Tm^3+^	Tm^3+^	Yb^3+^	980	264, 309	2008^156^
NaGdF_4_:Yb^3+^,Tm^3+^@NaYF_4_	Gd^3+^	Yb^3+^, Tm^3+^	980	310, 290	2011^160^
NaYF_4_@NaYbF_4_:Tm^3+^,Gd^3+^@NaYF_4_	Gd^3+^	Tm^3+^	980	290, 311, 350, 360	2016^161^
Rb_3_InCl_6_:Yb^3+^,Er^3+^	Er^3+^	Yb^3+^	980	384	2025^162^
NaGdF_4_:Yb^3+^,Tm^3+^@NaGdF_4_:Yb^3+^@NaGdF_4_:Yb^3+^,Nd^3+^@NaGdF_4_	Gd^3+^	Nd^3+^, Yb^3+^, Tm^3+^	808	253, 273, 276, 279, 306, 311	2021^163^
NaErF_4_@NaYF_4_	Er^3+^	Er^3+^	1550	382	2019^165^
NaYF_4_:Yb^3+^/Tm^3+^@NaErF_4_:Ce^3+^@NaYF_4_	Tm^3+^	Er^3+^, Yb^3+^	1550	290, 347, 362	2022^166^

On the other hand, by designing a core-shell structure to fine-tune the energy-transfer pathways, the impact of quenching centers on the crystal surface can be effectively minimized, and the ion doping concentration can be significantly increased, thereby achieving intense multiphoton UV upconversion luminescence^157–159^. Through energy transfer and migration between rare-earth ions, researchers have successfully realized UV upconversion luminescence under NIR laser excitation (808 nm, 980 nm, and 1550 nm), involving the following transitions: the ^4^G_11/2_→^4^I_15/2_ transition of Er^3+^ (382 nm), the ^1^I_6_→^3^H_6_ (290 nm) and ^1^I_6_→^3^F_4_ transitions of Tm^3+^ (350 nm), the ^6^P_7/2_→^8^S_7/2_ transition of Gd^3+^ (311 nm), and the 4f5d→^2^F_7/2_,^2^F_5/2_ transitions of Ce^3+^. Since 2011, Wang and colleagues have reported a series of core-shell structured UV upconversion luminescence nanoparticles, successfully eliminating deleterious cross-relaxation^160,161^. The NaGdF_4_:Yb^3+^/Tm^3+^@NaYF_4_ and NaYF_4_:Yb^3+^/Tm^3+^@NaYF_4_ core-shell nanostructures generate UV upconversion emissions upon 980 nm laser excitation. As illustrated in Fig. 10c, the emission at 310 nm confirmed efficient energy transfer from Tm^3+^ to Gd^3+^ within the core-shell nanoparticle. Recently, Zhang et al. reported intense UV emission at 384 nm in Yb^3+^/Er^3+^ co-doped Rb_3_InCl_6_, proposing a strategy to liberate the unusual ^4^G_11/2_→^4^I_15/2_ transition of Er^3+^ based on the confined energy transfer. Through this useful strategy to facilitate the UV upconversion luminescence of Er^3+^, the researchers have achieved the UV-to-green intensity ratio as high as 0.864, and the upconverted UV emission quantum yield of 0.12%^162^. Su et al. reported a multiphoton UV upconversion process in NaGdF_4_:Yb^3+^,Tm^3+^@NaGdF_4_:Yb^3+^@NaGdF_4_:Yb^3+^,Nd^3+^@NaGdF_4_ core-shell nanoparticles^163^. As shown in Fig. 10d, strong six-photon UV upconversion emission at 253 nm was realized upon 808 nm laser excitation. For UV emissions in the wavelength range of 240 to 400 nm, the quantum yield of upconversion luminescence is approximately 0.13%. This innovative design effectively minimizes energy dissipation caused by internal traps, enhances cascade sensitization from near-infrared excitation, and facilitates UVC luminescence originating from high-energy excited states.

In addition to the 980 and 808 nm lasers, a 1550 nm laser in the NIR-II region has also been used as an excitation source to achieve effective UV upconversion luminescence^164^. As shown in Fig. 10e, Sun et al. reported UV upconversion luminescence featuring a large Stokes shift of over 1150 nm, where Er^3+^ acts as both the sensitizer and activator in the upconversion process^165^. They utilized the high-energy integrated optical waveguide circuit to mitigate quenching of excited states, thereby enhancing the upconversion luminescence performance of the core-shell structured NaErF_4_@NaYF_4_ nanoparticle with overall energy conversion efficiency exceeding 5%. Shortly thereafter, they reported deep-UV upconversion luminescence at 290 nm with an ultra-large anti-Stokes shift of 1260 nm in NaYF_4_:Yb^3+^/Tm^3+^@NaErF_4_:Ce^3+^@NaYF_4_ core-shell-shell nanoparticles (Fig. 10f), in which a tandem upconversion process was effectively developed^166^.

### UV mechanoluminescence materials

As a distinct luminescence modality, mechanoluminescence (ML) has garnered considerable attention due to its real-time responsiveness to various mechanical stimuli, including friction, pressure, impact, crushing, and ultrasound^167^. Depending on the magnitude of the applied stress and the elastic threshold of the material, mechanoluminescent materials can be broadly categorized into three types: fracture-type, plastic-type, and elastic-type. Furthermore, based on whether pre-irradiation is required, these materials can be classified as either self-recovering or trap-controlled^168^. Each of these two ML types exhibits distinct advantages and disadvantages. Trap-controlled ML materials typically offer higher luminescence intensities but require inconvenient pre-irradiation treatment. In contrast, self-recovering ML materials are more attractive for practical applications owing to their ability to function without pre-irradiation. However, the range of available self-recovering materials remains limited, and their luminescence intensity is generally lower and in need of further enhancement. In recent years, UV ML has been progressively identified in certain UV-emitting material systems, as shown in Table 6. In most cases, these UV ML materials are incorporated into polymers to fabricate composite films or fibers, which aims to facilitate efficient force transmission.

**Fig. 11:**
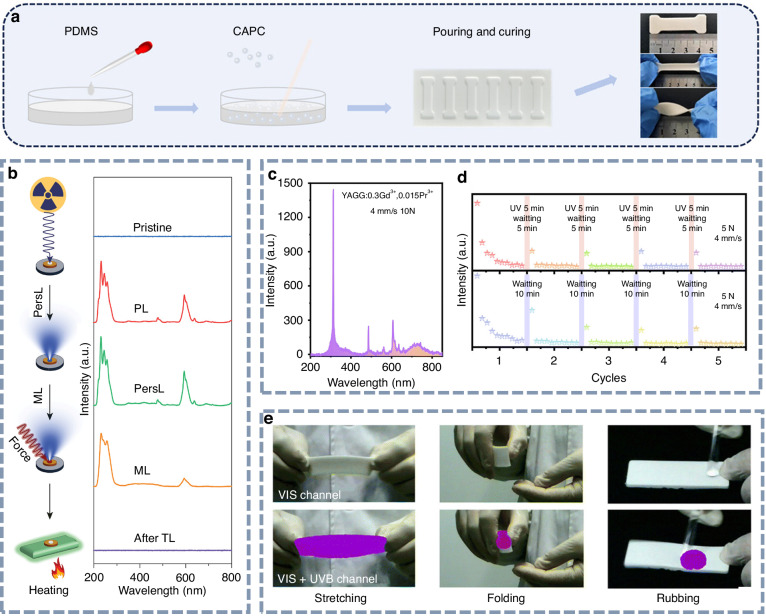
UV mechanoluminescence materials. **a** Schematic illustration of the fabrication process of mechanoluminescence composites. Reproduced with permission from ref. ^169^. Copyright 2025 John Wiley & Sons, Inc. **b** The process of trap-controlled mechanoluminescence. Reproduced with permission from ref. ^173^. Copyright 2024 John Wiley & Sons, Inc. **c** Mechanoluminescence spectrum and **d** mechanoluminescence recovery properties of the Y_3_Al_2_Ga_3_O_12_:Gd^3+^,Pr^3+^ film. Reproduced with permission from ref. ^174^. Copyright 2023 Royal Society of Chemistry. **e** Visible and UVB mechanoluminescence images of the ML film under the stimulation of stretching, folding, and rubbing. Reproduced with permission from ref. ^174^. Copyright 2023 Royal Society of Chemistry

Figure 11a illustrates the preparation process of a mechanoluminescent composite film using polydimethylsiloxane (PDMS) as the matrix^169^. The phosphor powder is homogeneously mixed with PDMS in a specific ratio, poured into a custom mold, and then cured at an appropriate temperature. Upon solidification, the composite film is demolded to obtain the desired shape. To date, most UV ML materials require a certain period of pre-irradiation and typically exhibit persistent luminescence characteristics^170,171^. In the initial stage of UV ML, Zhang et al. first reported trap-controlled UV ML from SrAl_2_O_4_:Ce^3+^ and SrAl_2_O_4_:Ce^3+^,Ho^3+^ phosphors. After the introduction of Ho^3+^, the concentration of shallow traps is remarkably enhanced, resulting in the improvement of UV ML intensity^172^. In 2024, Li et al. reported trap-controlled UVC ML in an X-ray charged Sr_2_P_2_O_7_:Pr^3+^ phosphor^173^. As illustrated in Fig. 11b, the phosphor generates UVC persistent luminescence following X-ray irradiation. After prolonged decay, the undetectable UVC signal can be reactivated by mechanical stimulation, yielding UVC ML.

Yang et al. developed a self-recovering UVB ML material, Y_3_Al_2_Ga_3_O_12_:Gd^3+^,Pr^3+^, where Pr^3+^ acts as a sensitizer to enhance the mechanoluminescence of Gd^3+^ ^174^. As shown in Fig. 11c, the composite film emits strong UVB ML with a peak at 313 nm when subjected to a mild tensile force of 10 N. After 10 consecutive stretching–releasing cycles, the emission intensity shows notable degradation, which can be partially restored following a 10-minute resting period (Fig. 11d). Using a UVB-sensitive camera, the invisible UVB ML can be captured. As illustrated in Fig. 11e, the UVB signals produced by various mechanical actions, such as stretching, folding, and rubbing, are visualized as distinct purple patterns. This high-performance, force-induced emission behavior offers a promising strategy for precise stress visualization and demonstrates significant potential in diverse UV applications. Cai et al. reported multi-responsive far-UVC luminescence at 222 nm in SrF_2_:Pr^3+^ phosphors, which exhibit both self-recovering and trap-controlled far-UVC ML under mechanical excitation/stimulation^175^. However, the trap-controlled ML was approximately 90 times more intense than the self-recovering counterpart, with the corresponding characterization of trap-controlled UVC ML presented. Consequently, the development of robust and self-recoverable UVC ML materials remains a formidable challenge in the field of photonics.

**Fig. 12:**
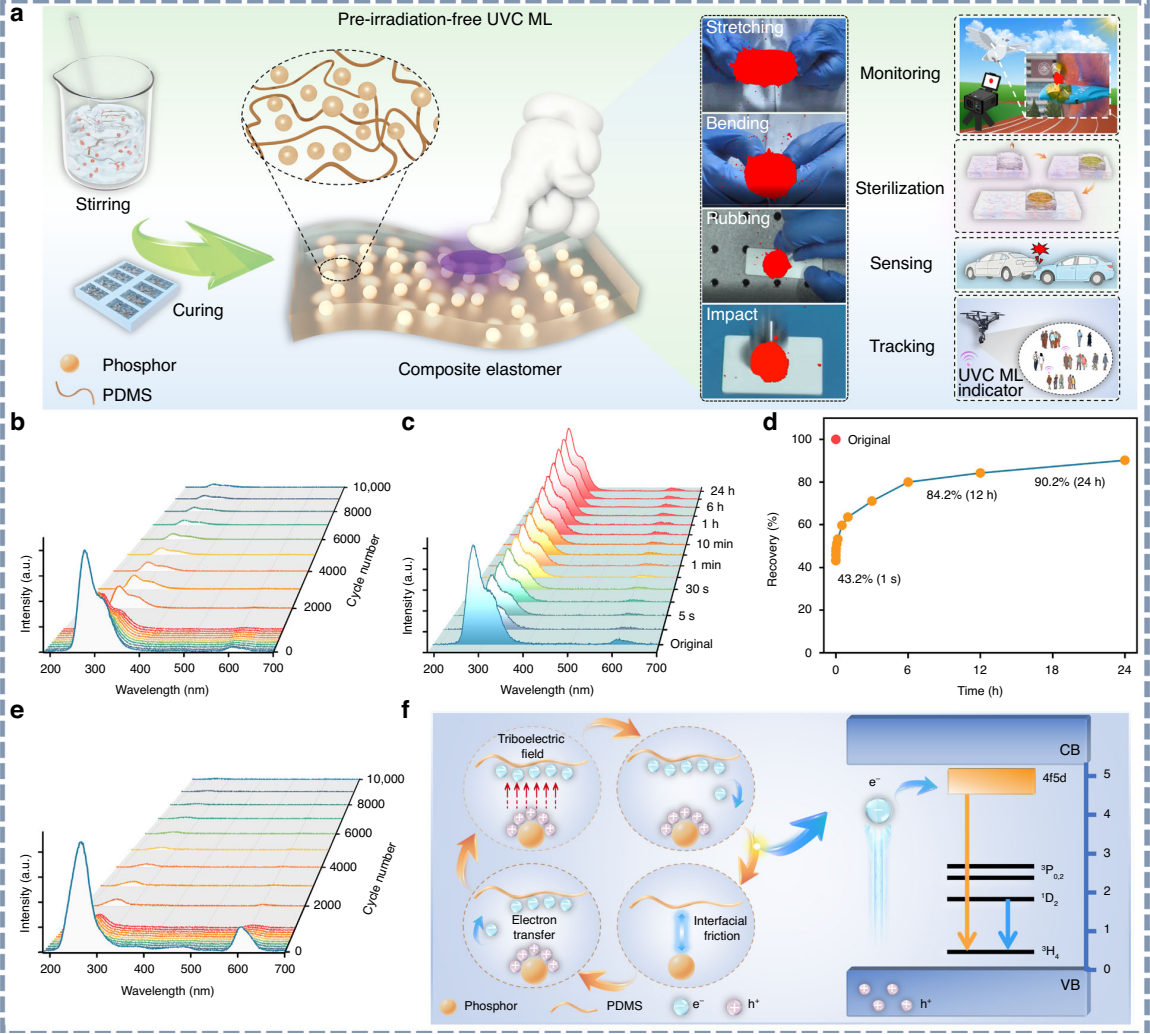
Pre-irradiation-free and self-recoverable UVC ML. **a** Schematic diagram of the synthesis, pre-irradiation-free UVC ML properties, and applications of the composite elastomer. Reproduced with permission from ref. ^177^. Copyright 2026 John Wiley & Sons, Inc. **b** UVC ML emission spectra of the Sr_3_(BO_3_)_2_:Pr^3+^/PDMS elastomer film over 10,000 continuous stretching cycles (frequency: 5 Hz; strain: 40%). Reproduced with permission from ref. ^176^. Copyright 2026 Springer Nature. **c** UVC ML emission spectra and **d** self-recovery evolution trend of the Sr_3_(BO_3_)_2_:Pr^3+^/PDMS elastomer film after being natural placed at room temperature for varying duration from 1 s to 24 h. Reproduced with permission from ref. ^176^. Copyright 2026 Springer Nature. **e** UVC ML emission spectra of the Sr_3_(PO_4_)_2_:Pr^3+^/PDMS composite elastomer over 10,000 continuous stretching cycles (test conditions: frequency 5 Hz; strain 40%). Reproduced with permission from ref. ^177^. Copyright 2026 John Wiley & Sons, Inc. **f** Schematic diagram of the potential self-powered UVC ML mechanism of the composite elastomer. Reproduced with permission from ref. ^177^. Copyright 2026 John Wiley & Sons, Inc

Most recently, Liang’s group reports a fundamentally new class of pre-irradiation-free, self-powered, and self-recoverable solar-blind UV mechanoluminescent elastomers by integrating their newly developed inorganic UVC phosphors into a flexible PDMS matrix, with representative ones including Sr_3_(BO_3_)_2_:Pr^3+^/PDMS^176^ and Sr_3_(PO_4_)_2_:Pr^3+^/PDMS^177^ (Fig. 12a). As shown in Fig. 12b–e, the obtained composite elastomers demonstrate excellent repeatability and cyclic stability, maintaining detectable UVC ML signal over 10,000 continuous stretching cycles. They also exhibit rapid and efficient self-recovery behavior. Combined experimental and theoretical analyses demonstrate that the observed UVC ML originates from a triboelectrification-dominated interfacial charge transfer mechanism (Fig. 12f). Leveraging the solar-blind nature and high photon energy of UVC light, these mechanoluminescent elastomers are well suited for all-weather practical applications such as structural failure monitoring, covert optical tagging and tracking, and advanced photonic applications beyond the visible spectrum.

**Table 6:** The detailed luminescence properties of UV mechanoluminescence materials

Material system	Mechanism	Emission peak (nm)	Ref.
SrAl_2_O_4_:Ce^3+^,Ho^3+^	Trap-controlled	375	2007^172^
LiYGeO_4_:Bi^3+^	Trap-controlled	355	2022^170^
NaYF_4_:Pr^3+^	Trap-controlled	250	2024^171^
Sr_2_P_2_O_7_:Pr^3+^	Trap-controlled	230	2024^173^
SrF_2_:Pr^3+^	Trap-controlled, self-recovered	222, 233, 259	2025^175^
Y_3_Al_2_Ga_3_O_12_:Gd^3+^,Pr^3+^	self-recovered	313	2023^174^
Ca_9_Al(PO_4_)_7_:Ce^3+^	self-recovered	344	2025^169^
Sr_3_(BO_3_)_2_:Pr^3+^	self-recovered	272	2026^176^
Sr_3_(PO_4_)_2_:Pr^3+^	self-recovered	264	2026^177^

## Emerging applications of inorganic UV luminescent materials

UV radiation is typically classified into three regions based on wavelengths: UVA, UVB, and UVC, each exhibiting distinct penetration depths and energy intensities. Due to the strong absorption of deep-UV radiation by the ozone layer, UVC light is entirely filtered out before reaching the Earth’s surface. Similarly, UVB radiation is largely blocked in indoor environments by common walls and glass materials. These wavelength-dependent characteristics of UV light impart unique functional properties, enabling their use in a wide range of applications. The following section provides a brief overview of the potential applications of inorganic UV-emitting materials, organized according to their emission wavelengths.

### UVA light for photocatalysis

**Fig. 13:**
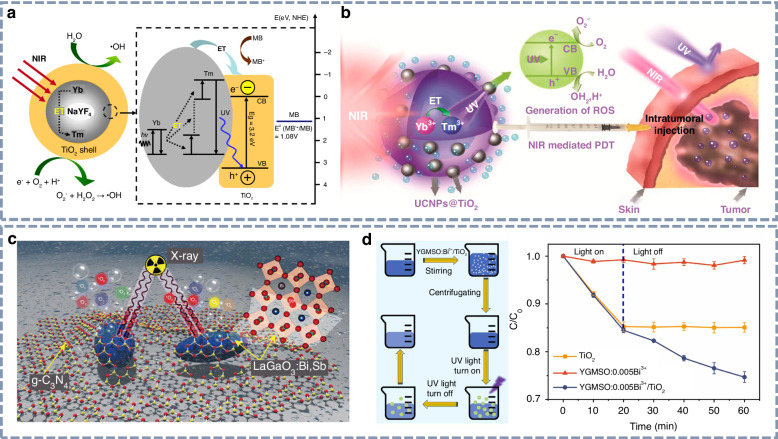
Potential applications of UVA light. **a** Schematic representation of the NIR-driven NaYF_4_:Yb^3+^,Tm^3+^@TiO_2_ core–shell upconversion nanoparticles for photocatalysis. Reproduced with permission from ref. ^178^. Copyright 2013 American Chemical Society. **b** Schematic representation of the NIR light-mediated NaYF_4_:Yb^3+^,Tm^3+^@NaGdF_4_:Yb^3+^ core–shell upconversion nanoparticles for in Vivo PDT. Reproduced with permission from ref. ^179^. Copyright 2015 American Chemical Society. **c** Schematic representation of the X-ray-activated PDT platform, LaGaO_3_:Bi^3+^,Sb^3+^@g-C_3_N_4_. Reproduced with permission from ref. ^84^. Copyright 2024 Royal Society of Chemistry. **d** Schematic representation of the photocatalytic degradation process and the methylene blue degradation curves. Reproduced with permission from ref. ^82^. Copyright 2024 Elsevier

UVA radiation, a form of long-wave UV light with relatively low energy, can induce photochemical reactions, making it highly valuable in a variety of applications such as optical anti-counterfeiting, fluorescence detection, and photocatalysis. Notably, photodynamic therapy (PDT)—a non-invasive and minimally damaging cancer treatment—has recently gained attention for its ability to destroy tumor cells by generating intracellular reactive oxygen species (ROS) through the photoactivation of photosensitizers. Titanium dioxide (TiO_2_), one of the most widely used photosensitizers, absorbs UV light to catalyze the production of ROS. As illustrated in Fig. 13a, Tang et al. employed NaYF_4_:Yb^3+^,Tm^3+^@TiO_2_ core-shell nanoparticles for photocatalysis under NIR irradiation^178^. The study demonstrated that energy transfer between the upconversion core NaYF_4_:Yb^3+^,Tm^3+^ and TiO_2_ shell plays a critical role in enhancing photocatalytic activity. The NIR-driven photocatalysis effectively promoted the degradation of organic pollutants by generating ROS. Hou et al. further developed a core-shell nanocomposite consisting of UV upconversion nanoparticles (NaYF_4_:Yb^3+^,Tm^3+^@NaGdF_4_:Yb^3+^) coated with TiO_2_ shell for NIR light-mediated PDT^179^. As shown in Fig. 13b, the efficient upconversion of NIR light into UV light was well-matched with the absorption spectrum of the TiO_2_ shell, providing a promising approach for achieving PDT in deep tissue regions.

In addition, the potential of UV afterglow materials as light sources for photocatalysis has also been demonstrated. As shown in Fig. 13c, a photodynamic therapy platform based on LaGaO_3_:Bi^3+^,Sb^3+^@g-C_3_N_4_ was constructed by leveraging the excellent UV persistent luminescence properties of LaGaO_3_:Bi^3+^,Sb^3+^ ^84^. This platform enables X-ray-activated PDT by integrating LaGaO_3_:Bi^3+^,Sb^3+^ phosphors with g-C_3_N_4_, utilizing the latter’s strong UV absorption characteristics. Moreover, Liu et al. explored the photocatalysis capabilities of Y_3_Ga_3_MgSiO_12_:Bi^3+^ phosphor^82^. The degradation of the methylene blue solution was used as a model reaction to assess photocatalytic performance. As presented in Fig. 13d, the Y_3_Ga_3_MgSiO_12_:Bi^3+^ phosphor exhibited effective photocatalytic degradation under both UV photoluminescence and UV persistent luminescence, offering a promising strategy for energy-efficient photocatalysis under dark conditions.

### UVB light for optical tagging, dermatological treatment, and information storage

UVB radiation, with a wavelength range of 280–320 nm, has attracted significant research interest due to its potential applications in dermatological therapies, photodynamic treatment, and covert indoor optical labelling. In indoor environments where natural UVB radiation is effectively blocked, particularly by walls and glass, UVB emissions that are invisible to the human eye can be captured in real time using UVB-sensitive cameras. This enables information encryption and interference-free optical signal transmission under standard indoor lighting conditions.

**Fig. 14:**
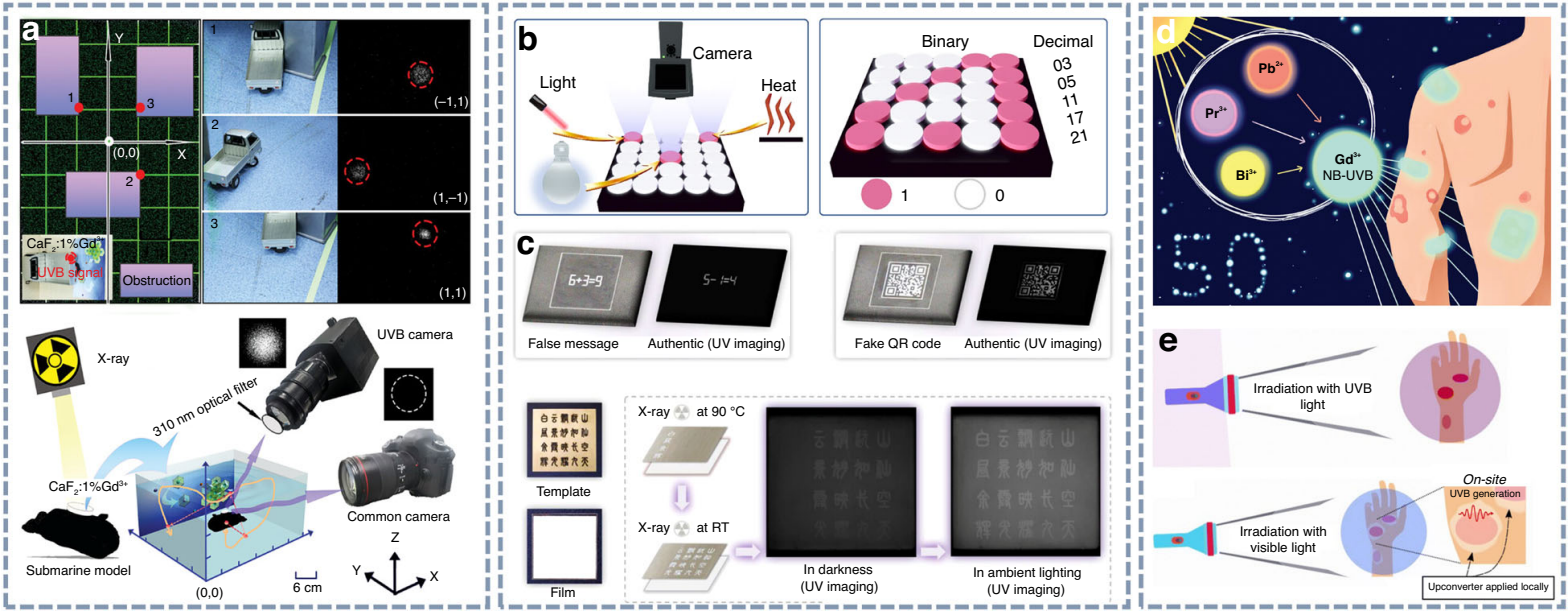
Potential applications of UVB light. **a** Static and dynamic tagging application of the X-ray-irradiated CaF_2_:Gd^3+^ phosphor. Reproduced with permission from ref. ^108^. Copyright 2024 John Wiley & Sons, Inc. **b** Optical information storage application of the X-ray-irradiated ScBO_3_:Bi^3+^ phosphor under light and heat stimulus. Reproduced with permission from ref. ^101^. Copyright 2024 Springer Nature. **c** Anti-counterfeiting application of the X-ray-irradiated NaYF_4_:Gd^3+^ phosphor under indoor lighting conditions. Reproduced with permission from ref. ^105^. Copyright 2025 John Wiley & Sons, Inc. **d** UVB phototherapy concept of the 254 nm-irradiated Sr_3_Gd_2_Si_6_O_18_:Pr^3+^ phosphor for small-area skin disorders. Reproduced with permission from ref. ^103^. Copyright 2021 Royal Society of Chemistry. **e** On-site UVB phototherapy application of the NaYF_4_:Ho^3+^, Gd^3+^ upconversion phosphor. Reproduced with permission from ref. ^180^. Copyright 2024 Royal Society of Chemistry

As illustrated in Fig. 14a, UVB afterglow signal can serve as tagging and tracking sources, demonstrating their feasibility in both static and dynamic tracking scenarios^107^. Moreover, UVB persistent phosphors have been employed in covert optical storage applications. For instance, ScBO_3_:Bi^3+^ (Fig. 14b) and NaYF_4_:Gd^3+^ (Fig. 14c) enable data encryption via ambient stimuli such as heat or light, which partially release trapped electrons and thus allow controlled readout of the stored information^100,104^. The hidden information can be accurately retrieved only with the aid of a UVB camera, ensuring high levels of security. Narrow-band UVB (NB-UVB) emissions, particularly in the 310–313 nm range, are known to modulate immune responses and have been validated as an effective strategy for treating various dermatological diseases. As shown in Fig. 14d, Gd^3+^-activated NB-UVB afterglow phosphors emit narrowband UVB luminescence centered around 312 nm and have been proposed for use in band-aid-like therapeutic patches^102^. These patches are portable, reusable, and customizable in both size and shape, allowing for localized and precision-targeted dermatological treatments. More recently, Yu et al. reported NB-UVB upconversion luminescence from NaYF_4_:Ho^3+^, Gd^3+^ nanoparticles, as depicted in Fig. 14e^180^. These nanoparticles can be excited by blue LED light to produce localized NB-UVB emissions, offering a promising strategy for precise and non-invasive phototherapy.

### UVC light for outdoor optical tagging and microbial sterilization

UVC radiation, with wavelengths ranging from 200 to 280 nm, falls within the short-wave ultraviolet region. Because UVC light is strongly absorbed by the ozone layer, it scarcely reaches the Earth’s surface and is therefore referred to as solar-blind UV radiation. Given that the emission spectra of solar-blind UVC light and ambient light are completely non-overlapping, UVC signals can be detected with high specificity using solar-blind UV cameras. This unique feature enables high-resolution UVC tagging and imaging in both indoor and outdoor environments, even under intense natural or artificial lighting conditions.

**Fig. 15:**
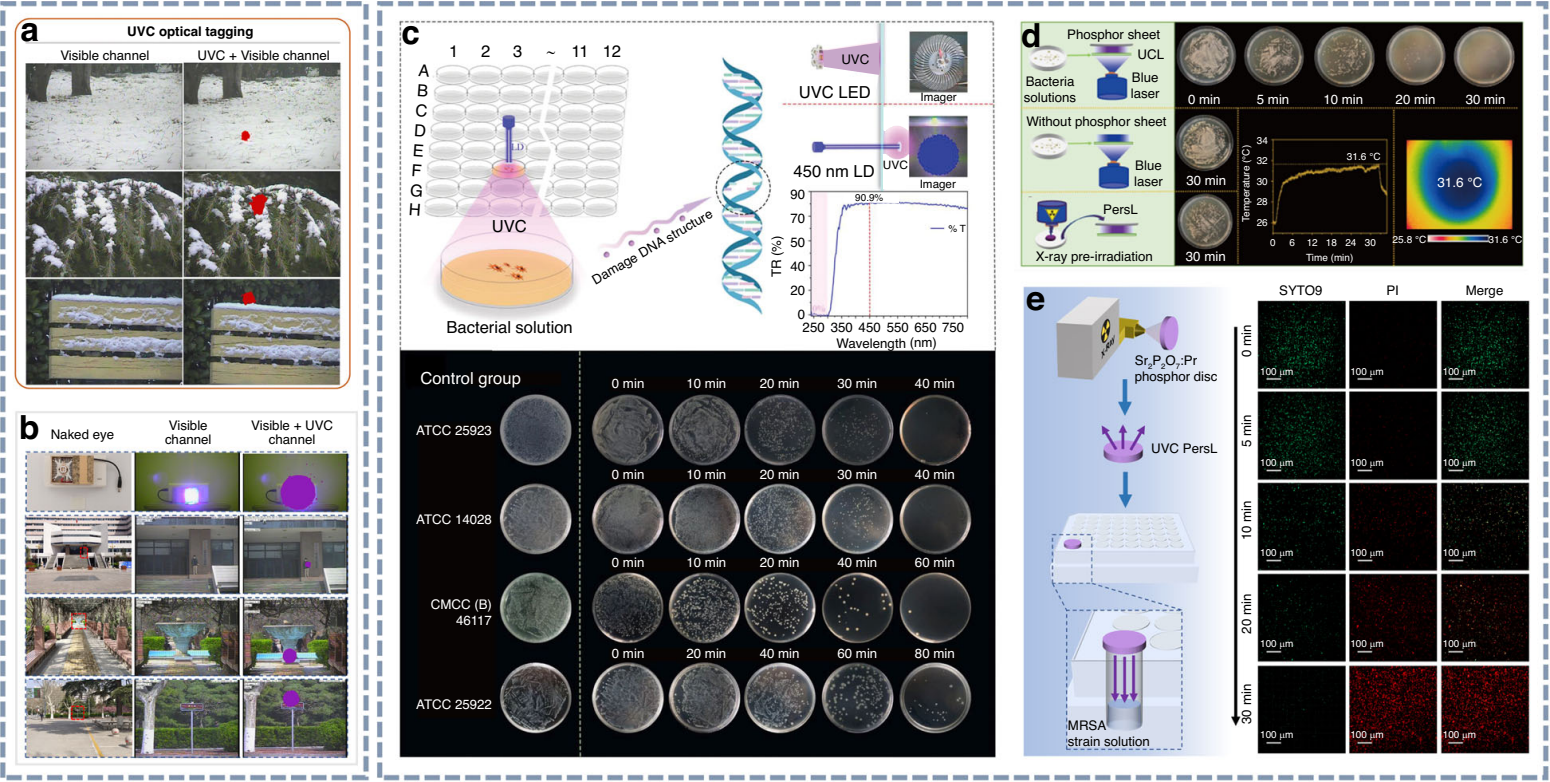
Potential applications of UVC light. **a** Optical tagging application of the X-ray-irradiated LiLuSiO_4_:Pr^3+^,Sm^3+^ phosphor on a snowy day. Reproduced with permission from ref. ^116^. Copyright 2022 Royal Society of Chemistry. **b** Optical tagging application of the Li_2_CaSiO_4_:Pr^3+^ upconversion phosphor upon 450 nm LED excitation. Reproduced with permission from ref. ^118^. Copyright 2023 Elsevier. **c** Sterilization application of the Li_2_SrGeO_4_:Pr^3+^ upconversion phosphor against *S*. *aureus*, *Salmonella enterica*, *Klebsiella pneumoniae*, and *E*. *coli*. Reproduced with permission from ref. ^146^. Copyright 2024 John Wiley & Sons, Inc. **d** Sterilization application of the multi-responsive Li_2_CaGeO_4_:Pr^3+^ phosphor against *S*. *aureus*. Reproduced with permission from ref. ^117^. Copyright 2021 Springer Nature. **e** Sterilization application of the X-ray-irradiated Sr_2_P_2_O_7_:Pr^3+^ phosphor against Methicillin-resistant *Staphylococcus aureus*. Reproduced with permission from ref. ^125^. Copyright 2023 Royal Society of Chemistry

As illustrated in Fig. 15a, the UVC afterglow of LiLuSiO_4_:Pr^3+^, Sm^3+^ phosphors can be monitored continuously at low temperatures when exposed to outdoor environments on a snowy day^116^. Additionally, remote UVC covert tagging has been demonstrated through UVC upconversion luminescence. As shown in Fig. 15b, a miniaturized UVC light source was realized through the combination of a 450 nm blue LED chip and a Li_2_CaSiO_4_:Pr^3+^-embedded PDMS composite film, successfully enabling optical tagging across a variety of complex outdoor scenarios^118^.

Beyond imaging and tagging, UVC light is known to disrupt the molecular structures of DNA and RNA, thereby offering efficient, broad-spectrum sterilization. This property has led to widespread applications of UVC light in medical and public health contexts. For example, Fig. 15c shows the bactericidal performance of Li_2_SrGeO_4_:Pr^3+^ phosphors, which emit UVC photons via blue-light-excited upconversion^146^. Upon irradiation, four types of bacteria were significantly inactivated, demonstrating the material’s potential for disinfection. Notably, this UVC emission can penetrate glass containers, making it suitable for sterilization in specialized environments. Figure 15d further exhibits a multi-responsive UVC phosphor capable of both upconversion luminescence and persistent luminescence^117^. These dual-mode emissions have been shown to effectively eliminate bacterial populations, underscoring the material’s utility in microbial control. Moreover, specific bands within the UVC range offer unique application advantages. In particular, far-UVC light has been demonstrated to inactivate pathogens such as bacteria and viruses while remaining safe for human skin and other tissues. As depicted in Fig. 15e, Liang’s group first developed a far-UVC persistent phosphor, Sr_2_P_2_O_7_:Pr^3+^, which effectively inactivated Methicillin-resistant *Staphylococcus aureus* after following X-ray charging^125^. This development presents a promising strategy for safe, excitation-free sterilization.

## Conclusions and outlook

This review provides a comprehensive examination of rare-earth and heavy main-group metal ion-doped inorganic UV phosphors, encompassing fundamental theoretical principles, material design, and potential applications. Particular emphasis is placed on three major categories of UV luminescence: persistent luminescence, upconversion luminescence, and mechanoluminescence. The evolution of UV-emitting materials over the past two decades has been systematically documented, highlighting key breakthroughs across these luminescence modalities. Additionally, the underlying mechanisms governing each type of UV emission are elucidated, offering guidance for the rational design and synthesis of next-generation UV phosphors.

Importantly, this work categorizes emerging UV-emitting technologies based on emission wavelength, enabling a structured and in-depth analysis of the current research landscape and future directions in UV persistent luminescence, upconversion luminescence, and mechanoluminescence systems. A critical link is also established between material properties and their functional applications, achieved through a comparative evaluation of UV-emitting materials across different spectral regions. Despite the considerable progress achieved, significant challenges remain, particularly in the areas of mechanistic understanding, strategic material design, performance enhancement, and practical integration. Future research should prioritize addressing these challenges to facilitate the transition of inorganic UV phosphors from fundamental scientific inquiry to real-world application.

1. **Optimization of guiding principles for UV luminescent material design**: Compared to well-characterized luminescent centers, host matrices offer greater flexibility and thus broader design possibilities. While energy band engineering has provided foundational paradigms for material design, most existing UV luminescent systems remain confined to relatively narrow compositional spaces. Future advancement will rely on the establishment of comprehensive UV luminescence databases and the integration of artificial intelligence (AI)-driven materials discovery frameworks. Such approaches are expected to significantly reduce the cost of experimental iteration and facilitate the rational design of phosphors with tailored emission characteristics.
2. **Development of UV-emitting materials with low-threshold excitation**: Most currently UV-emitting materials require high-energy excitation sources, such as X-rays for UV persistent luminescence or high-power lasers for UV upconversion luminescence, which hinders their practical deployment. The development of next-generation UV phosphors capable of low-threshold excitation under readily available sources—such as commercial blue LEDs—requires the strategic optimization of non-radiative energy transfer pathways and enhancement of absorption cross-sections. Achieving efficient UV upconversion or upconverted persistent luminescence under low-power excitation would mark a significant breakthrough in the field. For example, Liu’s group recently reported a pioneering approach to achieving ultraviolet (UV) emission via phonon-assisted anti-Stokes luminescence under visible-light excitation in a variety of phosphors, including LuBO_3_:Ce^3+^, LiLuGeO_4_:Bi^3+^, and NaYF_4_:Nd^3+^, providing new insights into phonon-assisted UV luminescence through the use of visible light^181^.
3. **Efficiency enhancement and spectral control of inorganic UV phosphors**: In comparison to commercial UV-emitting alternatives, rare-earth or heavy main-group metal ion-doped inorganic UV phosphors often exhibit lower quantum efficiencies and offer limited tunability, particularly in the deep-UV region (<250 nm). These performance limitations restrict their industrial applicability. Advancing materials that provide both high luminescence efficacy and precise wavelength control, especially within the germicidal UVC range, is critical to realizing the full potential of features such as intrinsic “self-luminescence” for practical applications. Notably, to circumvent the inherent limitations of low emission intensity (or quantum yield) in UV upconversion phosphors, several advanced strategies have been proposed. Specifically, the integration of core–shell architectures, precise optimization of sensitizer concentrations, the utilization of surface plasmon resonance, and the deliberate crystal lattice engineering have emerged as effective routes for performance enhancement.
4. **Multi-stimuli responsive luminescence modulation**: UV persistent luminescence, especially in deep-trap systems, is notably responsive to external stimuli such as optical and thermal inputs, where ambient light can enhance afterglow intensity. Expanding this concept, future research should explore UV phosphors that respond to a broader array of stimuli, including mechanical, electrical, and magnetic fields. The development of such multidimensionally responsive UV luminescent materials—particularly mechanoluminescent systems—holds promise for significantly broadening the scope of UV-based technologies in imaging, sensing, security, and biomedical applications.

## Data Availability

All the data supporting the findings of this study are presented within the article. Additional data related to this paper are available from the corresponding authors upon request. Source data are provided within this paper.
